# Identification of B-cell epitopes of Indian Zika virus strains using immunoinformatics

**DOI:** 10.3389/fimmu.2025.1534737

**Published:** 2025-02-27

**Authors:** Rohan Raj Roy, Nitali Tadkalkar, Gururaj Rao Deshpande, Nitin M. Atre, Pratip Shil, Gajanan Sapkal

**Affiliations:** ^1^ Diagnostic Virology Group, Indian Council of Medical Research (ICMR) - National Institute of Virology, Pune, India; ^2^ Bioinformatics and Data Management, Indian Council of Medical Research (ICMR) - National Institute of Virology, Pune, India

**Keywords:** Zika virus, immunoinformatics, B-cell epitopes, neutralization, monoclonal antibodies, molecular docking, complementary determining regions, 2-D interaction maps

## Abstract

**Introduction:**

The Zika virus is an emerging Flavivirus known to cause Zika infection in humans. It is associated with severe health problems such as microcephaly and Guillain-Barré syndrome post the Brazilian epidemic in 2015-16. The spread of the Zika virus to the Asian subcontinent, especially to India is a matter of great concern. Two recent co-circulating Indian Zika virus strains such as Rajasthan and Maharashtra detected in 2018 and 2021 were studied to identify B-cell epitopes in the envelope and non-structural 1 protein as these epitopes are major indicators of robust humoral immune response. The study aimed at identifying novel epitopes, followed by molecular docking with potent Zika virus-specific monoclonal antibodies. The novel epitopes identified in this study shall be essential in designing multi-epitope vaccines capable of inducing antibody response against Zika virus infection.

**Methods:**

ABCpred, BepiPred 2.0 and Kolaskar-Tongaonkar methods were used for predicting the linear B-cell epitopes, and Discotope 2.0 and ElliPro were used for the prediction of conformational epitopes. Linear epitopes were further checked for protective antigenicity, allergenicity and toxicity. Based on the stringent study design criteria, only the novel epitopes were considered for molecular docking with complementary determining regions of potent Zika virus-specific monoclonal antibodies.

**Results:**

Nineteen linear and five conformational epitopes were shortlisted based on protective potential, non-allergic and non-toxic properties for Zika virus E protein, from which nine linear and three conformational epitopes were identified as novel. Molecular docking studies revealed that the novel linear epitopes, one each from EDIII, EDII, EDI and EDI/DIII hinge were involved in epitope-CDR interactions with potent neutralizing Zika virus E-specific mouse monoclonal antibody ZV-67. Moreover, the novel EDII epitope was exclusively engaged in epitope-CDR interactions of potent neutralizing Zika virus E-specific human monoclonal antibody Z3L1. None of the linear epitopes of Zika virus NS1 were ascertained as novel based on our study criteria. Conformational epitopes were identified as novel for NS1 protein.

**Conclusion:**

This study identified Zika virus-specific novel epitopes of envelope and non-structural -1 proteins in the currently co-circulating Indian strains. Furthermore, in-silico validation through molecular docking added insight into antigen-antibody interactions, paving way for future *in vitro* and *in vivo* studies.

## 1 Introduction

Zika virus (ZIKV) infection is an emerging Flavivirus transmitted by the *Aedes* mosquitoes, mainly *Aedes aegyptii* and *Aedes albopictus*. ZIKV originated in Africa and was first isolated from the serum of rhesus macaque at the Zika forest in Uganda in 1947 (1). Over the decades, ZIKV infection prevailed in the African continent and South Asia, happening sporadically with minimal symptoms and mild illness due to which it was not considered a serious health problem until the first major outbreak in 2007 in the Yap islands in Micronesia (2), followed by French Polynesia in 2013-14 (3). It was a matter of serious concern when the ZIKV accumulated various mutations and spread to South America through Brazil in late 2014 which caused a major outbreak associated with microcephaly and birth defects in newborns and infants (4). In 2016, there was a 20-fold increase of ZIKV infection cases in Brazil with around 304 cases of microcephaly (4) Moreover, ZIKV infection was also implicated in causing Guillain–Barré syndrome (GBS) (5). ZIKV also spread to Central America particularly, the United States. Therefore, the World Health Organization (WHO) declared ZIKV infection a Public Health Emergency of International Concern in 2016 (6, 7). The ZIKV surveillance was started in India in March 2016 through the network of Virus Research Diagnostic Laboratories (VRDLs) by the Indian Council of Medical Research (ICMR), following which sporadic cases of ZIKV were found in Gujarat (2016–17) and Tamil Nadu (2017). In late 2018, one hundred fifty-nine and one hundred twenty-seven ZIKV cases were reported in Rajasthan and Madhya Pradesh, respectively (8–10). The identification of the Rajasthan strain led to the first laboratory confirmation of the existence of the Zika Virus in India (8). Moreover, in the year 2021, Maharashtra reported their first cases of Zika virus infections which were confirmed in Belsar village in Pune district (11). Since then, Zika virus cases have expanded to Kerala, Uttar Pradesh and 16 states of India which is a matter of concern as currently there is no universal vaccine or antivirals. The symptoms of ZIKV are self-limited and resolve usually between 2-7 days. Small subgroups of patients may develop serious complications like GBS that require hospitalization and monitoring of mechanical ventilation, intravenous immunoglobulin and electrophoresis (12–14).

Humoral immune response to ZIKV infection is one of the major ways to accomplish protective immunity regulated by B-cells. ZIKV-specific B-cells are activated in response to infection which secrete IgG and IgA antibodies post-IgM antibodies produced during the acute phase of infection (15). Antigen-antibody interaction studies are important in understanding the immune response in viral infections (16, 17). B-cell epitopes are crucial for understanding protective immunity as well as immunopathogenesis (18). Neutralizing antibodies are mostly produced against ZIKV envelope (E) proteins which tend to identify and bind to specific B-cell epitopes to elicit robust protection (19). The most potent neutralizing antibodies bind to B-cell epitopes on the E domain III (EDIII) which is the least conserved region among different ZIKV strains. Hence, EDIII-specific epitopes are regarded as ZIKV type-specific (20). The other domains such as E domain I (EDI) and E domain II (EDII) are generally regarded as ZIKV/DENV and Flavivirus cross-reactive domains, respectively and epitopes of these domains also induce protective immunity to ZIKV (21). Among the non-structural proteins, Non-structural 1 (NS1) is the most enigmatic protein of the *Flaviviruses.* ZIKV NS1 has a multifunctional role in viral replication, pathogenesis and immune evasion (22). Recently, ZIKV NS1 has been seen as a potential vaccine candidate as it contains epitopes targeted by ZIKV-specific monoclonal antibodies (mAbs) (23, 24). The NS1 protein exists in two forms, a dimer or/and a hexamer (secretory NS1). Similar to the E protein, NS1 also possess three important domains such as β-roll, wing domain with three subdomains (α/β subdomain, long intertwined loops and discontinuous connector subdomain), and β-ladder which also contains the spaghetti loop (25, 26). Most ZIKV-NS1-specific mAbs, target epitopes on the wing-domain and β-ladder domains. To be precise, the epitopes which are a part of the exposed and outer surface of NS1, such as the spaghetti loop residues of the β-ladder and the first half of the intertwined loop of the wing domain are the most effective targets of the antibody response (26). However, the other NS1 domains and subdomains may also contain certain epitopes which may be of potential research interest regarding ZIKV infection and inhibition.

The emergence of immuno-informatics allows the use of various prediction tools and software to compare and analyze various aspects of virus-induced immune response in a less time-consuming, and cost-effective manner. As a result, various computational methods have been used to predict potential B-cell epitopes for arboviruses (16, 17, 27, 28).

In this study, we have undertaken a domain-specific approach to identify and compare the ZIKV E and NS1 B-cell epitopes in both the Indian ZIKV strains which are currently in co-circulation using the epitope prediction tools, present at the immune epitope database (IEDB; http://tools.iedb.org/main/bcell/). We have predicted both linear and conformational B-cell epitopes, the former composed of residues continuous in the sequence and the latter being distantly separated in the sequence but possessing spatial closeness. The epitopes predicted in this study have been compared with the known ZIKV-specific B-cell neutralizing epitopes enlisted in the IEDB. Epitopes with zero per cent identity with known ZIKV-specific neutralizing epitopes (IEDB) were considered novel. Subsequently, these epitopes were mapped to the three-dimensional (3-D) structures of the E and NS1 proteins of both the Indian ZIKV strains. Then these were compared to the prototype African ZIKV MR766 (African lineage) and the Brazilian ZIKV Natal RGN (Asian lineage) associated with microcephaly. Finally, these novel epitopes were used to study their binding interactions with complementary determining regions (CDRs) of ZIKV-specific highly neutralizing monoclonal antibodies (mAbs) by molecular docking analysis. The resulting 3-D and two-dimensional (2-D) interaction maps were used to select the novel epitopes possessing intermolecular bonding interactions such as conventional hydrogen (H) bonds with the CDRs of the neutralizing mAbs. This shall be essential in designing ZIKV-specific peptides for Indian ZIKV strains.

## 2 Materials and methods

### 2.1 ZIKV E and NS1 protein sequence retrieval and antigenicity prediction

The polyprotein sequences of both the Indian ZIKV strains i.e. ZIKV Rajasthan (ZIKV_RAJ; GenBank ID: AZS35409.1) and ZIKV Maharashtra (ZIKV_MAH; GenBank ID: UBI73854.1) were obtained from the NCBI protein database. The polyprotein sequences of the prototype African strain (ZIKV MR766; GenBank ID: YP 009227198.1) and the Brazilian ZIKV strain associated with microcephaly (ZIKV Natal RGN; GenBank ID: YP 009428568.1) were used as a reference for comparison. The amino acid sequences of the E and NS1 proteins of these ZIKV were extracted from the polyprotein sequences and subjected to antigenicity prediction in the VaxiJen version 2.0 (29). This server used an alignment-free approach for antigen prediction based on auto cross-covariance (ACC) transformation of protein sequences into uniform vectors of principal amino acid properties.

### 2.2 Domain-specific antigenicity prediction

The retrieved ZIKV E and NS1 sequences were subjected to multiple sequence alignment using MEGA11 with ClustalW and MUSCLE alignment algorithms. The domain-wise antigenicity of the E and NS1 proteins of ZIKV_RAJ and ZIKV_MAH were calculated with the help of VaxiJen v2.0 having a threshold of 0.4. These antigenicity scores were also compared with the antigenicity of ZIKV MR766 and ZIKV Natal RGN.

### 2.3 Three-dimensional structure prediction

3-D structures of these proteins were predicted by homology modelling protocols as defined in MODELLER v10. 4. The templates for the E and NS1 proteins of ZIKV_RAJ and ZIKV_MAH were searched at NCBI using Protein Data Bank (PDB) via Position-Specific Iterated BLAST (PSI-BLAST). The PDB IDs which had the highest query cover (~100%), per cent identity (~100%) and lowest E values (=0) with the target sequences were selected as the templates. All 3-D models generated were validated via SAVES SERVER (https://saves.mbi.ucla.edu/) using PROCHECK. The best model was selected considering the amino acid occupancies in Ramachandran plots.

### 2.4 Domain-specific linear B-cell epitope prediction

Linear B-cell epitope prediction for both E and NS1 proteins was carried out by using ABCpred, BepiPred 2.0 and Kolaskar & Tongaonkar methods. ABCpred mediated epitope prediction used a machine-learning-based artificial neural network algorithm, whereas the IEDB B cell epitope prediction tools such as BepiPred 2.0 and Kolaskar Tongaonkar methods were based on random forest algorithm and semi-empirical antigenicity, respectively (30–32). The common epitope sequences from the three methods were considered for further analysis, with a minimal length ranging from 5-25 amino acids for each predicted epitope (33). To determine the exposed and buried residues, the surface accessibility of these epitopes was calculated using the Emini surface accessibility scale (34) and the hydrophilicity of the epitopes was determined by Parker hydrophilicity (32). Based on all the above parameters, the list of predicted B-cell epitopes specific to ZIKV E and NS1 was tabulated. Multiple sequence alignments of the proteins of ZIKV_RAJ, ZIKV_MAH, ZIKV MR766 and ZIKV NATAL RGN strains were used for comparative analysis (**Supplementary Figures S1**, **S2**). The ZIKV-specific B-cell epitopes of the E and NS1 proteins aligned to the respective domains were individually checked for being antigenic, non-allergic and non-toxic by Vaxijen v2.0 (29, 35–37), respectively.

### 2.5 Prediction of novel and overlapping domain-specific linear B-cell epitopes

The IEDB epitope database was searched for all the linear B-cell neutralizing epitopes of ZIKV. The search strategy for ZIKV-specific epitopes was linear epitopes with exact matches of the organism Zika virus (ID:64320); host as Homo sapiens (human) (ID:9606), Mus musculus (mouse) (ID:10090), and Mus musculus C57BL/6 (ID:10000067, c57) and the filter was set as “B-cell neutralization; biological activity (neutralization)” to obtain all the ZIKV-specific neutralizing epitopes submitted at IEDB till date. The predicted ZIKV E-domain specific epitopes were compared individually with these IEDB-neutralizing epitopes using BLAST~70% to determine overlapping (BLAST~70% positive) and non-overlapping (BLAST~70% negative) epitopes. The non-overlapping epitopes were further checked for per cent identity based on amino acid composition with the known IEDB-neutralizing epitopes to determine the novel epitopes (Zero per cent identity).

To identify overlapping and non-overlapping ZIKV NS1-domain specific epitopes, the search strategy in IEDB was similar to that of the E epitopes, except for the filter, which was set as “B-cell antibody binding (any method)”. Similarly, all the predicted ZIKV NS1-domain specific epitopes were compared individually with the ZIKV NS1 IEDB epitopes using Blast~70%.

### 2.6 Prediction of domain-specific conformational B-cell epitopes

The conformational B-cell epitopes were predicted by two IEDB-based methods i.e. Discotope 2.0 and ElliPro (38, 39). Three-dimensional structures were used as input for epitope prediction. Discotope 2.0 predicted the epitopes based on their solvent accessibility, contact numbers and propensity scores while ElliPro used surface protrusion, accessibility and flexibility.

### 2.7 Prediction of novel and overlapping domain-specific conformational B-cell epitopes

The IEDB epitope database was searched for all the conformational B-cell epitopes of ZIKV. The search strategy was linear epitope prediction as “discontinuous epitopes with exact matches”, and the filter selected as “B-cell neutralization; biological activity (neutralization)” for both ZIKV E and NS1 protein. Epitopes which were not identical post-comparison with IEDB-neutralizing discontinuous epitopes were considered novel and the others were regarded as overlapping or identical.

### 2.8 Mapping and visualization of predicted epitopes to the ZIKV E and NS1 domains

BIOVIA Discovery Studio Visualizer is a free, feature-rich molecular modelling application for viewing, sharing and analyzing protein and small molecule data. All the relevant epitopes predicted were mapped onto their protein structure in their respective domains and visualized through the BIOVIA Discovery Studio Visualizer

### 2.9 Molecular docking of ZIKV E and NS1-specific novel epitopes with monoclonal antibodies

The ZIKV 3-D models of E and NS1 were subjected to molecular docking with the 3-D structures of highly neutralizing ZIKV mAbs by using HDock (http://hdock.phys.hust.edu.cn/) which incorporates a hybrid-docking algorithm (40). The best models were selected by analyzing their receptor-ligand interface residues required to identify epitope-CDR interactions. Among the top 10 models, the most suitable epitope-CDR interactions were selected by comparing all the relevant 3-D and 2-D receptor-ligand interaction maps in BIOVIA Discovery Studio Visualizer.

### 2.10 Study design

**Figure 1:**
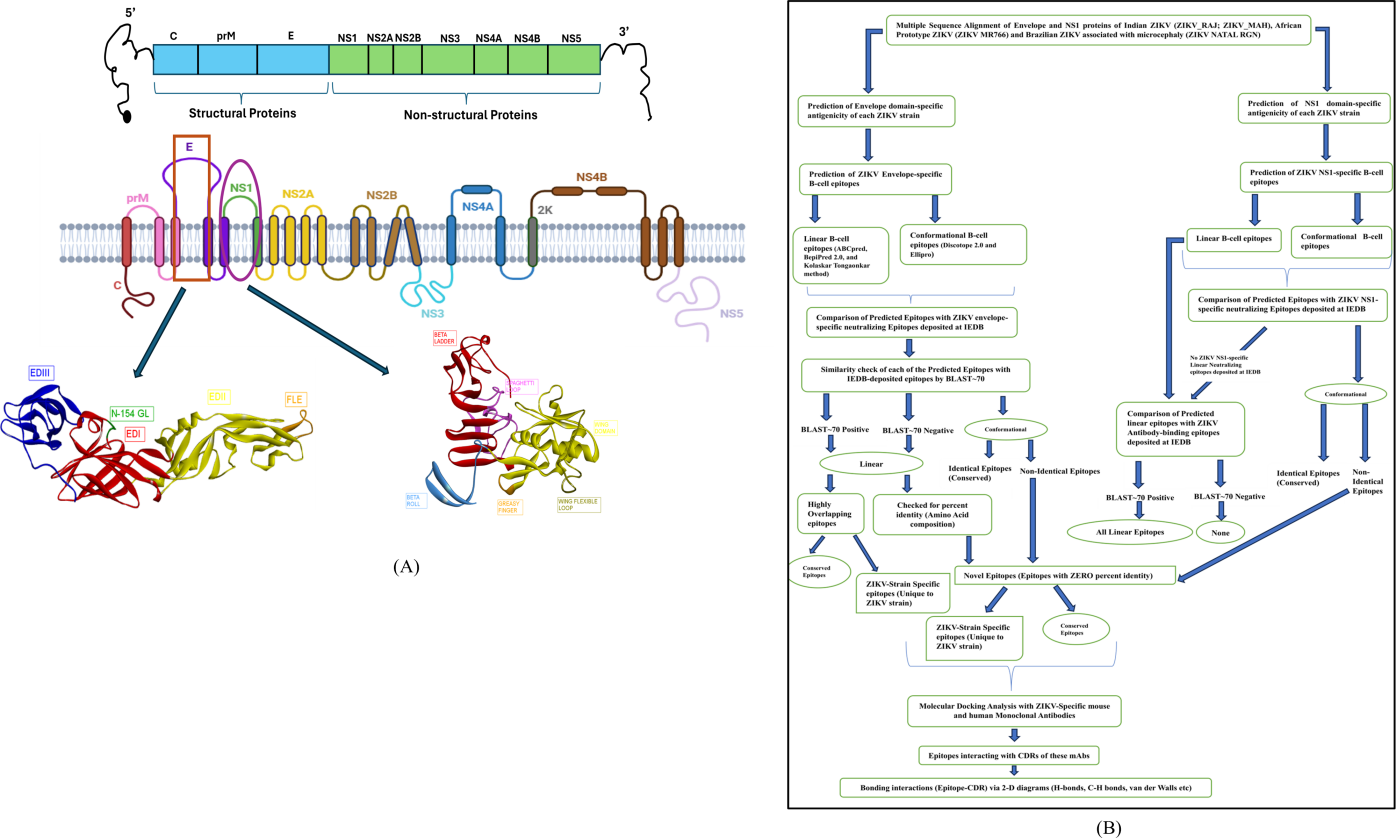
Schematic illustration and presentation of the study. **(A)** ZIKV genome highlighting the E and NS1 proteins, **(B)** Study design criteria.

The overall study design is depicted in the following **Figure 1**.

## 3 Results

### 3.1 Protective antigenicity of both E and NS1 proteins of ZIKV

The predicted antigenicity scores of the E protein of both ZIKV_RAJ and ZIKV_MAH were found to be 0.6268 and 0.6417, respectively using VaxiJen version 2.0 (29). Similarly, the E proteins of ZIKV MR766 and ZIKV Natal RGN had antigenicity scores of 0.6276 and 0.6205, respectively. The same methodology was used for predicting the protectiveness of the ZIKV NS1 proteins calculated as ZIKV_RAJ (0.4487); ZIKV_MAH (0.4630); ZIKV MR766 (0.4455); and ZIKV Natal RGN (0.4607) Both the ZIKV E and NS1 proteins were considered as protective antigens with the E protein having higher antigenic scores than NS1.

### 3.2 Delineation and comparisons of ZIKV domain-specific antigenicity of E and NS1

The ZIKV E protein consisted of three domains namely domains 1,2 and 3 (EDI, EDII and EDIII), respectively. The positions of these three domains were mapped on the E protein of the ZIKV strains (41) (**Supplementary Table S1**). In the case of NS1, similarly, three different domains were identified as β-Roll, Wing-domain, and β-ladder, and their positions were mapped accordingly (25) (**Supplementary Table S2**). To dissect the domain-specific epitopes of the Indian ZIKV, it was necessary to evaluate the antigenicity of the respective domains (**Supplementary Tables S1**, **S2**). This gave a comparative analysis of the domains as well as differences in antigenicity between the ZIKV strains. These values were also compared with the antigenicity of ZIKV MR766 and ZIKV NATAL RGN strains.

It was evident from **Supplementary Table S1** that all the domains of the E protein are antigenic with a minor non-antigenic region in EDII (52–131) in the case of both the Indian strains and Brazilian ZIKV NATAL RGN, unlike the African ZIKV MR766. Moreover, EDII was highly conserved across the ZIKV strains demonstrated by identical antigenicity scores., EDI had varying antigenicity scores, except positions (1–50, 39) which had identical antigenicity across all ZIKV strains. EDI (132–191) and EDI (280–295) had different antigenicity scores for both Indian ZIKV strains. The EDIII (296–403) had varying antigenicity scores for two ZIKV strains (ZIKV MR766 and ZIKV_MAH) and identical scores for the other two ZIKV strains (ZIKV_RAJ and ZIKV NATAL RGN), which corroborates this region as ZIKV type-specific (39).

**Supplementary Table S2** summarizes the antigenicity analyses for NS1. The β-Roll domain was antigenic with identical scores for both the Indian ZIKV strains and the ZIKV NATAL RGN, unlike African ZIKV MR766. The wing-domain antigenicity scores varied among the Indian, Brazilian and African ZIKV strains and were found to be below the threshold of 0.4, identifying it as non-antigenic. However, the wing domain of ZIKV NS1 contributes to monoclonal antibody-mediated protection, hence epitopes of this region are of research interest (22). The β-ladder domain had varying antigenicity scores with ZIKV_MAH being the highest, followed by ZIKV NATAL RGN, ZIKV_RAJ and ZIKV MR766.

Overall, our analyses revealed that the domain-specific antigenicity of the E and NS1 proteins of Indian ZIKV was differential as well as identical with the African and Brazilian ZIKV strains. Moreover, similarities and differences in the antigenic domains were also observed between the Indian strains ZIKV_RAJ and ZIKV_MAH.

### 3.3 3D structure prediction of Indian ZIKV E and NS1 proteins

**Figure 2:**
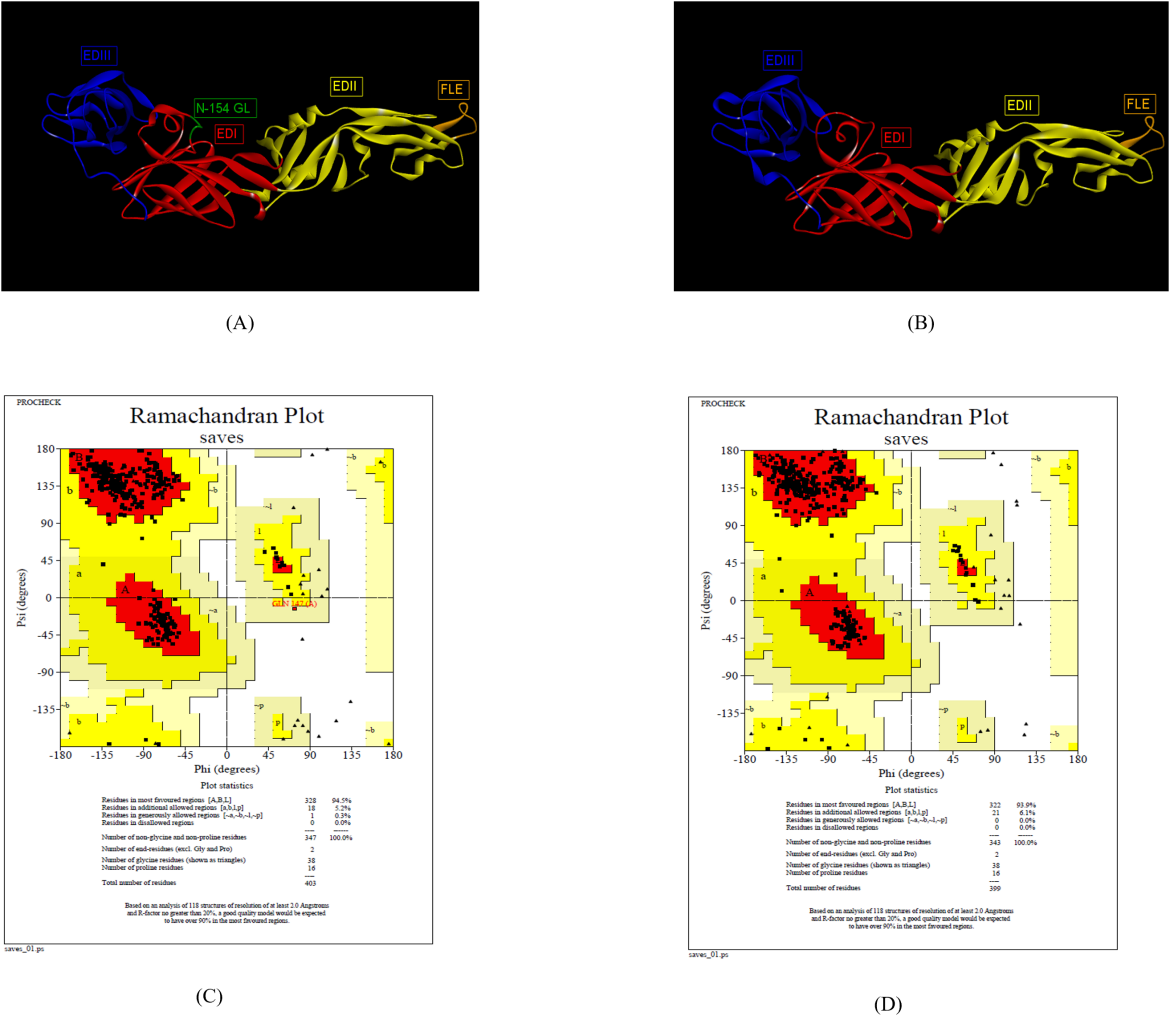
Reliable 3-D models for both the Indian ZIKV E and their validation. **(A)** ZRE (Predicted model for ZIKV_RAJ E protein) along with its Dope score **(B)** ZME (Predicted model for ZIKV_MAH E protein) along with its Dope score, **(C)** Ramachandran plot results of ZRE and **(D)** ZME.

The 3-D structure ZIKV E protein ectodomain ZIKV_RAJ (1–403) and ZIKV_MAH (1–399) were predicted with the crystal structures of ZIKV E protein as templates: 7YW8.pdb (**Supplementary Figure S3A**) and 7YW7.pdb (**Supplementary Figure S4B**), respectively. Similarly, the templates for ZIKV MR766 and ZIKV NATAL RGN were identified as 7YW7.pdb and 7YW8.pdb, respectively. The best models were selected based on Ramachandran plot analysis: ZRE (ZIKV_RAJ; **Figure 2A**) and ZME (ZIKV_MAH; **Figure 2B**). The occupancy of amino acids in the most favorable and additionally allowed regions for ZRE (**Figure 2C**) was 99.7% (94.5% + 5.2%) and for ZME (**Figure 2D**) was 100% (93.1% and 6.1%). There was not a single amino acid occupying the disallowed regions in both the predicted structures. One striking difference was the presence of the N-154 (154-NDTG-157) glycan loop in ZRE (**Figure 2A**) and its deletion in ZME (**Figure 2B**). The N-linked glycosylation was also evident in ZIKV NATAL RGN and absent in ZIKV MR766. These results indicated differences in the E-protein structures of both the Indian ZIKV strains.

**Figure 3:**
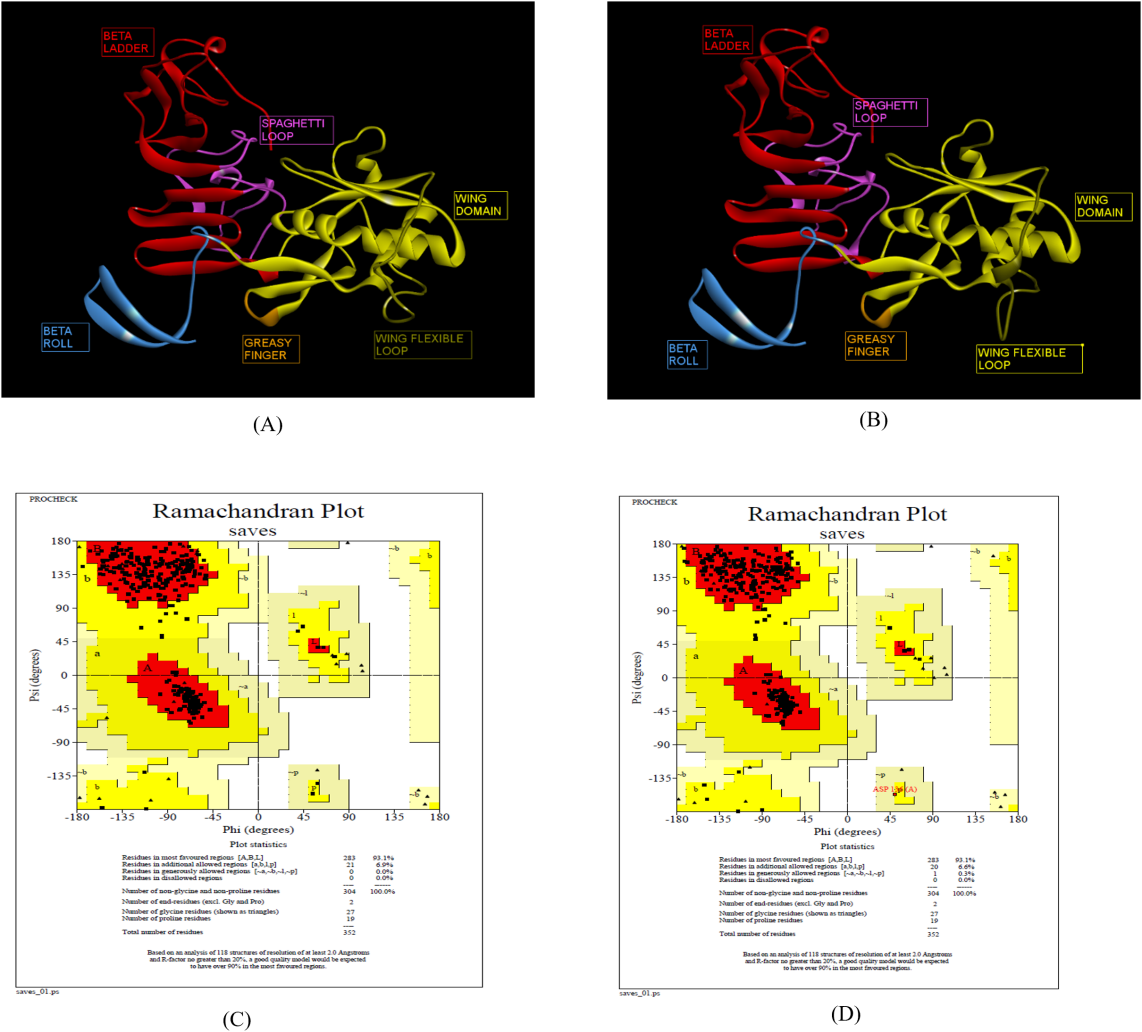
Reliable 3-D models for both the Indian ZIKV NS1 and their validation. **(A)** ZRNS1 (Predicted model for ZIKV_RAJ NS1 protein) along with its Dope score, **(B)** ZMNS1 (Predicted model for ZIKV_MAH NS1 protein) along with its Dope score, **(C)** Ramachandran plot results of ZRNS1 and **(D)** ZMNS1.

The 3D structures of ZIKV NS1 (Indian, African and Brazilian strains) were predicted using the known structure of ZIKV NS1 (5K6K.pdb; **Supplementary Figure S4**) (25). **Figure 3** shows the predicted structures of NS1 for the Indian strains: ZRNS1 (ZIKV_RAJ; **Figure 3A**) and ZMNS1 (ZIKV_MAH; **Figure 3B**). ZRNS1 and ZMNS1 both had zero per cent residues in disallowed regions. The occupancy of amino acids in the most favored and allowed regions was found to be 100% (93.1% + 6.9%) and 99.7% (93.1% + 6.6%) for ZRNS1 (**Figure 3C**) and ZMNS1 (**Figure 3D**).

### 3.4 Prediction of ZIKV E-domain specific linear epitopes

**Figure 4:**
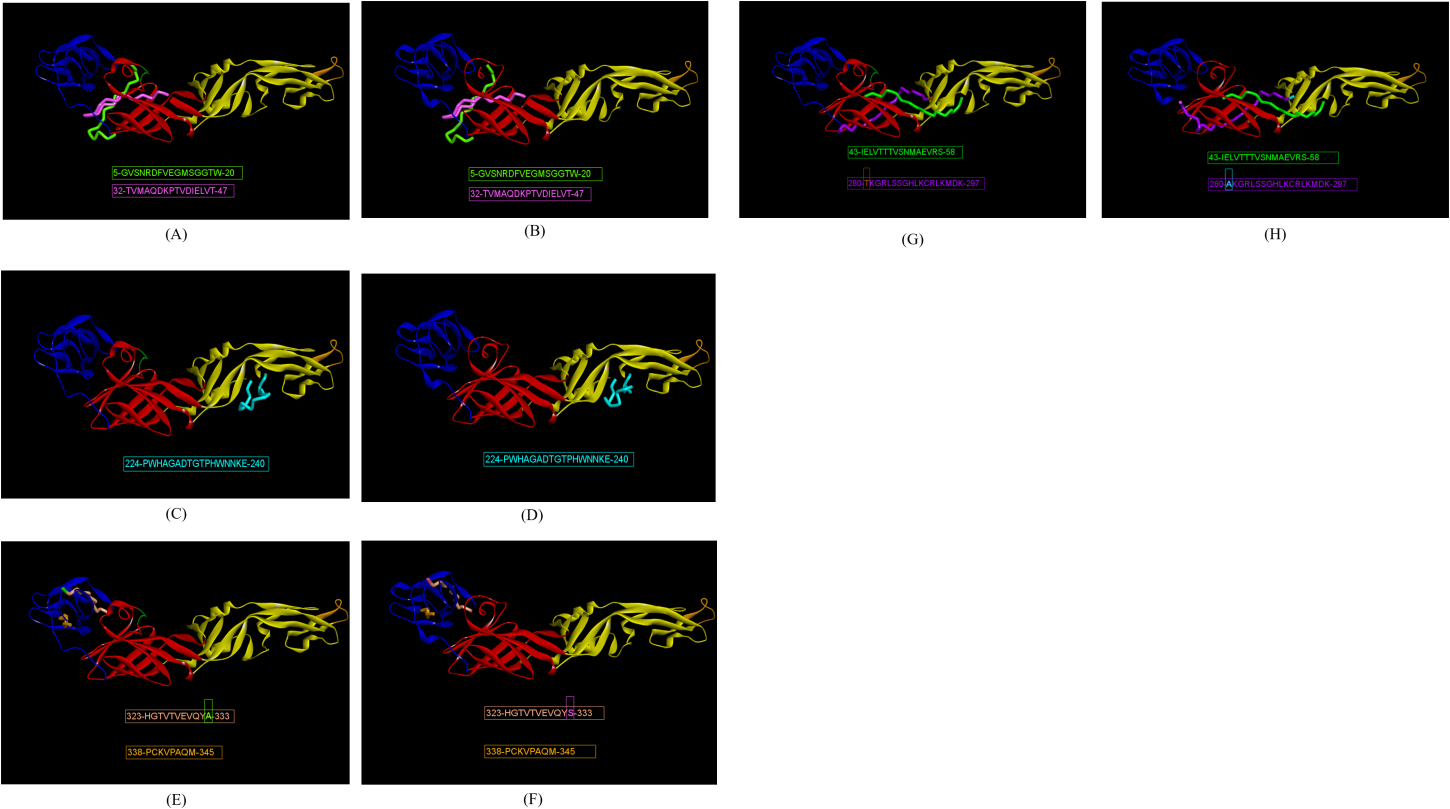
Indian ZIKV E domain-specific novel linear epitopes. **(A)** ZRE domain I-specific epitopes, **(B)** ZME domain I-specific epitopes, **(C)** ZRE domain II-specific epitopes, **(D)** ZME domain II-specific epitopes, **(E)** ZRE domain III-specific epitopes, **(F)** ZME domain III-specific epitopes, **(G)** ZRE hinge region-specific epitopes, and **(H)** ZME hinge region-specific specific epitopes.

The prediction of E-domain-specific epitopes was done with a combination of three immunoinformatic tools such as ABCpred, BepiPred 2.0 and Kolaskar-Tongaonkar methods. Twenty-eight and twenty-five epitopes were predicted for ZIKV_RAJ and ZIKV_MAH, respectively via ABCpred with a threshold of 0.8 (**Supplementary Table S3**). In the case of BepiPred 2.0, 18 epitopes were predicted for ZIKV_RAJ and 22 for ZIKV_MAH at 0.5 as threshold (**Supplementary Figure S4**, **Supplementary Table S4**). Furthermore, epitope predictions by Kolaskar and Tongaonkar methods revealed 20 and 19 epitopes for ZIKV_RAJ (threshold= 1.026) and ZIKV_MAH (threshold=1.028), respectively (**Supplementary Figure S5**, **Supplementary Table S5**). Exposed and buried epitopes were predicted by Emini surface accessibility methods, where scores above 1.00 were considered positive (**Supplementary Figure S6**). The hydrophilic stretches were determined by Parker hydrophilicity predictions with thresholds of 1.701 and 1.726 for ZIKV_RAJ and ZIKV_MAH, respectively (**Supplementary Figure S7**). Overlapping shortlisted epitopes were further scanned for being potentially antigenic, non-toxic and non-allergic which identified 33 epitopes for ZIKV E protein (**Table 1**). To determine the non-overlapping and overlapping epitopes, the predicted epitopes were compared with the IEDB database for ZIKV-specific B-cell epitopes involved in neutralizing antibody response. It revealed two non-overlapping epitopes 5-GVSNRDFVEGMSGGTW-20 and 32-TVMAQDKPTVDIELVT-47 (**Figures 4A, B**) which had zero percentage identity with the IEDB neutralizing epitopes. Hence, these were designated as novel epitopes having antigenic scores 0.589 and 0.624, respectively. Both these epitopes were conserved across ZIKV_RAJ and ZIKV_MAH. Moreover, there was no amino acid mutation when these epitopes were compared with ZIKV MR766 and ZIKV Natal RGN. However, the other epitope 165-AKVEVTPNSPRAEATL-180 was present in ZIKV MR766 with an antigenic score of 0.6917 but a single point mutation from valine to isoleucine 165-AKVEITPNSPRAEATL-180 (V169I) in Indian ZIKV (ZIKV_RAJ and ZIKV_MAH) and ZIKV Natal RGN increased its antigenicity to 0.8044. These epitopes 165-AKVEVTPNSPRAEATL-180 and 165-AKVEITPNSPRAEATL-180 had a 12.5% per cent identity with the IEDB neutralizing epitopes and were considered overlapping epitopes.

**Table 1:** ZIKV E domain-specific linear B-cell epitopes prediction.

Epitope sequence	ZIKV E-specific domain	Antigenicity	Allergenicity	Toxicity
5-GVSNRDFVEGMSGGTW-20 32-TVMAQDKPTVDIELVT-47 165-AKVEVTPNSPRAEATL-180 165-AKVEITPNSPRAEATL-180	EDI	Antigenic 0.589 Antigenic 0.624 Antigenic 0.691 Antigenic 0.804	Non-allergen Non-allergen Non-allergen Non-allergen	Non-Toxin Non-Toxin Non-Toxin Non-Toxin
61-YEASISDMASDSRCPT-76 98-DRGWGNGCGLFGK-110 197-DFSDLYYLTMNNKHWL-212 224-PWHAGADTGTPHWNNKE-240	EDII	Antigenic 0.435 Antigenic 0.486 Antigenic 1.171 Antigenic 1.154	Non-allergen Non-allergen Allergen Non-allergen	Non-Toxin Non-Toxin Non-Toxin Non-Toxin
323-HGTVTVEVQYA-333 323-HGTVTVEVQYS-333 338-PCKVPAQM-345 338-PCKIPVQM-345 363-PVITESTENSK-373 363-PVITESAENSK-373 384-DSYIVIGVGDKKITHHWHRS-403 384-DSYIVIGVGEKKITHHWHRS-403	EDIII	Antigenic 1.219 Antigenic 1.256 Antigenic 0.450 Antigenic 0.478 Antigenic 0.634 Antigenic 0.452 Antigenic 0.920 Antigenic 0.844	Non-allergen Non-allergen Non-allergen Non-allergen Non-allergen Non-allergen Non-allergen Non-allergen	Non-Toxin Non-Toxin Non-Toxin Non-Toxin Non-Toxin Non-Toxin Non-Toxin Non-Toxin
43-IELVTTTVSNMAEVRS-58 185-SLGLDCEPRTGLD-197 118-KFTCSKKMTGKSIQPE-133 118-KFACSKKMTGKSIQPE-133 126-TGKSIQPENLEYRIMLSV-143 280-AKGRLSSGHLKCRLKMDK-297 280-TKGRLSSGHLKCRLKMDK-297 280-AKGRLFSGHLKCRLKMDK-297	Hinge-Regions	Antigenic 0.813 Antigenic 1.048 Antigenic 0.423 Non-Antigenic 0.358 Antigenic 1.088 Antigenic 0.488 Antigenic 0.566 Non-Antigenic 0.026	Non-allergen Allergen Non-allergen Non-allergen Non-allergen Non-allergen Non-allergen Non-allergen	Non-Toxin Non-Toxin Non-Toxin Non-Toxin Non-Toxin Non-Toxin Non-Toxin Non-Toxin

Mutations of amino acids are colored with respect to the African prototype strain (ZIKV MR766; colored in green and corresponding mutations are colored in red; Novel Epitopes are underlined).

There were four EDII-specific B-cell epitopes, out of which 61-YEASISDMASDSRCPT-76 and 98-DRGWGNGCGLFGK-110 were antigenic with scores as 0.435 and 0.486, respectively. 98-DRGWGNGCGLFGK-110 was considered a fusion loop epitope (FLE) as it spanned across the fusion loop domain. The other epitopes 197-DFSDLYYLTMNNKHWL-212 and 224-PWHAGADTGTPHWNNKE-240 had high antigenicity scores of 1.1716 and 1.1547, respectively, but the former was found to be an allergen and was excluded from the analysis. The epitope 224-PWHAGADTGTPHWNNKE-24 (**Figures 4C, D**) had zero per cent identity with IEDB neutralizing epitopes, hence considered as novel. The epitope 61-YEASISDMASDSRCPT-76 was non-overlapping (BLAST~70% negative), but had 25% per cent identity with the neutralizing epitopes at IEDB, hence was not regarded as novel. The FLE 98-DRGWGNGCGLFGK-110 was BLAST~70% positive with the IEDB neutralizing epitopes and considered as overlapping. Moreover, all the EDII-specific epitopes were conserved across ZIKV_RAJ, ZIKV_MAH, ZIKV MR766 and ZIKV NATAL RGN.

The highest number of ZIKV-specific linear B-cell epitopes were predicted for EDIII. eight epitopes were predicted, harboring point mutations across the ZIKV strains. Out of them, four epitopes, 323-HGTVTVEVQYA-333 (**Figure 4E**) and 323-HGTVTVEVQYS-333 (**Figure 4F**) as well as 338-PCKVPAQM-345 (**Figure 4E**) and 338-PCKIPVQM-345 (**Figure 4F**) were non-overlapping and had zero per cent identity with IEDB neutralizing epitopes, indicating these as novel. Epitope 323-HGTVTVEVQYA-333 was found in ZIKV_RAJ, ZIKV MR766 and ZIKV NATAL RGN, whereas 323-HGTVTVEVQYS-333 having A333S mutation was found exclusively in ZIKV_MAH. A333S mutation had increased the antigenicity from 1.2198 to 1.2569. Epitope 338-PCKIPVQM-345 was found only in ZIKV MR766 while 338-PCKVPAQM-345 was identified across both the Indian ZIKV (ZIKV_RAJ, ZIKV_MAH) and ZIKV Natal RGN with mutations I341V and V343A leading to increase in antigenicity from 0.450 to 0.478. The remaining epitopes, 363-PVITESTENSK-373 present in ZIKV_RAJ, ZIKV MR766 and ZIKV Natal RGN and 363-PVITESAENSK-373 (ZIKV_MAH), were BLAST~70%-positive and considered overlapping. In contrast, to the novel epitope mutations, the T369A mutation in epitope 363-PVITESAENSK-373 found only in ZIKV_MAH resulted in decreased antigenicity from 0.92 to 0.84. The epitope 384-DSYIVIGVGDKKITHHWHRS-403 in ZIKV_MAH, and 384-DSYIVIGVGEKKITHHWHRS-403 in ZIKV_RAJ were BLAST~70%-negative but had 10% identity with IEDB-neutralizing epitopes. Hence, these were not considered as novel. D393E mutation was also associated with decrease in antigenicity scores from 0.63 to 0.45. Moreover, 393D was found in ZIKV MR766 and 393E in ZIKV NATAL RGN. These EDIII epitopes with point mutations across the ZIKV strains are important for studying type-specific antibody responses to ZIKV infection.

The predicted B-cell linear epitopes also spanned across the hinge regions of the E protein as these regions are important in potently cross-neutralizing Flavivirus infections, especially ZIKV and DENV infections. A total of eight hinge region epitopes were predicted with epitope 126-TGKSIQPENLEYRIMLSV-143 (conserved in both Indian strains) having the highest antigenicity of 1.0884, followed by epitope 43-IELVTTTVSNMAEVRS-58 (conserved in both Indian strains) with antigenicity of 0.8137, both of which spanning EDI/II hinge and conserved in the Indian strains. Out of these two epitopes, 43-IELVTTTVSNMAEVRS-58 (**Figures 4G, H**) was BLAST~70% negative and had zero per cent identity with the IEDB neutralizing epitopes, hence regarded as novel. The other epitope 126-TGKSIQPENLEYRIMLSV-143 was BLAST~70% positive, overlapping with IEDB neutralizing epitopes. Epitope 118-KFTCSKKMTGKSIQPE-133 was found in ZIKV MR766 but the T120A mutation in both Indian ZIKV strains and ZIKV NATAL RGN led to a decrease in antigenicity from 0.42 to 0.35. These epitopes were BLAST~70% positive and considered overlapping epitopes. Moving ahead, the epitope 280-AKGRLFSGHLKCRLKMDK-297 in ZIKV MR766 was found to be highly non-antigenic with a score of 0.02, but the A280T and F285S mutations in 280-TKGRLSSGHLKCRLKMDK-297 (ZIKV_RAJ; **Figure 4G**) reversed its non-antigenicity, making it antigenic with a score of 0.56. Similarly, the F285S mutation in 280-AKGRLSSGHLKCRLKMDK-297 (ZIKV_MAH; **Figure 4H**) also made it antigenic with a score of 0.48. Both these epitopes, unique to Indian ZIKV strains were BLAST~70%-negative and had zero per cent identity with the IEDB neutralizing epitopes, indicating these as novel EDI/DIII epitopes. The epitope 185-SLGLDCEPRTGLD-197 of the EDI/II hinge region was antigenic but was excluded from the analysis due to its allergenicity. The change in antigenicity based on the mutations among the epitopes of the hinge regions would be essential to studying peptide-specific reactivity to ZIKV and related Flavivirus infections such as DENV.

#### 3.4.1 ZIKV NS1 domain-specific linear B-cell epitopes

The prediction of linear B-cell epitopes for the NS1 protein of ZIKV was carried out in the same manner as that of the E protein. Following the E protein, the ZIKV antibody response is triggered by the NS1 protein. Hence, it is necessary to identify ZIKV NS1 domain-specific epitopes. The immunoinformatic tools for the prediction of linear B-cell epitopes of NS1 were the same as those used for the ZIKV E. ABCpred predicted a total of 24 and 26 epitopes with a threshold of 0.8 for ZIKV_RAJ and ZIKV_MAH, respectively (**Supplementary Table S6**). BepiPred 2.0 predicted 11 epitopes for each Indian ZIKV (ZIKV_RAJ and ZIKV_MAH) with a threshold of 0.5 (**Supplementary Table S7**, **Supplementary Figure S7**). Kolaskar and Tongaonkar method predicted 16 epitopes for each Indian ZIKV (ZIKV_RAJ and ZIKV_MAH) with a threshold of 1.018 and 1.023 for ZIKV_RAJ and ZIKV_MAH, respectively (**Supplementary Table S8**, **Supplementary Figure S8**). Similar to the E protein, these epitopes were checked for accessibility and hydrophilicity by Emini surface accessibility and Parker hydrophilicity, respectively (**Supplementary Figures S9**, **S10**). In total, sixteen epitopes were predicted for ZIKV occupying different NS1-specific domains (**Table 2**). Among them, nine epitopes were found in Indian ZIKV strains. Most of the epitopes predicted possessed point mutations among them and were ZIKV strain-specific which may be interesting to study via *in vitro* and *in vivo* peptide validation experiments.

**Table 2:** ZIKV NS1 domain-specific linear B-cell epitopes prediction.

Epitope sequence	ZIKV NS1-specific domain	Antigenicity	Allergenicity	Toxicity
10-KKETRCGTGVFIYNDVE-26 10-KKETRCGTGVFVYNDVE-26	β-Roll	Antigenic 0.780 Antigenic 0.875	Non-allergen Non-allergen	Non-Toxin Non-Toxin
83-GVQLTVVVGSVKNP-96 83-GIQLTVVVGSVKNP-96 141- ECPLEHRAWNSFLVED-157 141- ECPLKHRAWNSFLVED-157 141- ECPLKHRAWNSFIVED-157	Wing Domain	Antigenic 0.748 Antigenic 0.820 Antigenic 0.587 Antigenic 0.505 Antigenic 0.487	Allergen Non-allergen Non-allergen Non-allergen Non-allergen	Non-Toxin Non-Toxin Toxin Non-Toxin Non-Toxin
191- REAAHSDLGYWIESEKND-208 191- KEAVHSDLGYWIESEKND-208 248-AGPLSHHNTREGYRTQV-264 248-AGPLSHHNTREGYRTQM-264 331-YGMEIRPRKEPESNLVRSMV-350 331-YGMEIRPRKEPESNLVRSVV-350	β-Ladder	Antigenic 0.430 Antigenic 0.572 Antigenic 0.713 Antigenic 0.802 Antigenic 0.623 Antigenic 0.680	Non-allergen Allergen Non-allergen Non-allergen Non-allergen Non-allergen	Non-Toxin Non-Toxin Non-Toxin Non-Toxin Non-Toxin Non-Toxin
176-SLECDPAVIGTAVKGREAA-194 176-SLECDPAVIGTAVKGKEAV-194 176-SLECDPAVIGTAIKGKEAV-194	Wing-β-Ladder	Antigenic 1.173 Antigenic 1.093 Antigenic 1.087	Non-allergen Non-allergen Non-allergen	Non-Toxin Non-Toxin Non-Toxin

Mutations of amino acids are colored with respect to the African prototype strain (ZIKV MR766; colored in green and corresponding mutations are colored in red).

The epitope 10-KKETRCGTGVFVYNDVE-26 in the beta roll domain of NS1 was conserved across both the Indian ZIKV strains (ZIKV_RAJ and ZIKV_MAH). This epitope was also conserved in the ZIKV Natal RGN. However, the ZIKV MR766 strain had I21 instead of V21. This I21V mutation in the rest of the strains resulted in increased antigenicity from 0.78 to 0.87.

The exposed surfaces of NS1 are composed of the wing domain, especially the residues except for the flexible loop, greasy finger. Considering this, five epitopes were predicted for the wing domain. All these linear epitopes were associated with point mutations. The epitope 83-GVQLTVVVGSVKNP-96 was specific to both African ZIKV (ZIKV MR766) and Brazilian ZIKV (ZIKV Natal RGN) but V84I mutation in the same epitope 83-GIQLTVVVGSVKNP-96 was specific to both the Indian ZIKV strains (ZIKV_RAJ and ZIKV_MAH). This epitope in African and Brazilian strains was predicted as an allergen, but the V84I mutation in the Indian strains made the epitope non-allergen (by AllerTop). This mutation also increased the antigenicity from 0.74 to 0.82. Similarly, another set of predicted epitopes as 141-ECPLEHRAWNSFLVED-157 was specific to ZIKV MR766 and ZIKV Natal RGN. Point mutations were seen in both the Indian ZIKV strains such as E145K and L153I (ZIKV_RAJ) and only L153I (ZIKV_MAH). The E145K mutation in ZIKV_MAH was associated with decreased antigenicity from 0.58 to 0.50 while both E145K and L153I mutations in ZIKV_RAJ led to a further decrease in antigenicity to 0.48. Interestingly, the epitope 141-ECPLEHRAWNSFLVED-157 specific to ZIKV MR766 and ZIKV Natal RGN was predicted to be toxic (ToxinPred), whereas in the Indian strains, the epitopes 141-ECPLKHRAWNSFIVED-157 (ZIKV_RAJ) and 141-ECPLKHRAWNSFIVED-157 were found to be non-toxic.

The beta ladder is another domain of NS1 which is a target for ZIKV NS1-specific neutralizing mAbs as it is mostly exposed, especially the spaghetti loop residues. The highest number of predicted epitopes belonged to this domain. The epitope 191- REAAHSDLGYWIESEKND-208 in ZIKV MR766 possessed mutations: R191K and A194V in both Indian strains. This resulted in an increase in antigenicity from 0.43 to 0.57. Though there was an increase in antigenicity, the epitope 191- KEAVHSDLGYWIESEKND-208 was found to be an allergen (by AllerTop) and was not considered for further analysis. Another epitope 248-AGPLSHHNTREGYRTQV-264 was conserved in ZIKV_MAH and ZIKV_MR766 while the epitope 248-AGPLSHHNTREGYRTQM-264 was conserved in ZIKV_RAJ and ZIKV NATAL RGN. This V264M mutation in ZIKV_RAJ was associated with an increase in antigenicity from 0.71 to 0.80. The epitope 331-YGMEIRPRKEPESNLVRSMV-350 was conserved in ZIKV MR766 and ZIKV RAJ whereas the M349V mutation observed in ZIKV_MAH and ZIKV Natal RGN was associated with an increase in antigenicity from 0.62 to 0.68.

Moving forward, the epitope 176-SLECDPAVIGTAVKGVEAA-194 present in ZIKV MR766 was highly antigenic (antigenicity=1.17; VaxiJen) This was located as part of the connector residues linking the wing domain to the beta ladder domain. Mutations V188I, R191K, and A194V were observed in the ZIKV_MAH strain while mutations R191K and A194V were observed in ZIKV_RAJ with respect to ZIKV MR766. Epitope 176-SLECDPAVIGTAVKGKEAV-194 in ZIKV_RAJ and ZIKV Natal RGN had an antigenicity of 1.09 and 176- SLECDPAVIGTAIKGKEAV-194 in ZIKV_MAH had an antigenicity of 1.08.

According to our study design, the IEDB ZIKV-neutralizing epitopes database did not contain any linear B-cell ZIKV NS1-specific epitope. Comparing the predicted ZIKV NS1-specific B-cell linear epitopes with the IEDB ZIKV-antibody binding epitopes revealed that all these B-cell epitopes were BLAST~70% positive. This suggests that all the predicted linear epitopes of the ZIKV NS1 overlap with linear ZIKV NS1-specific antibody-binding epitopes. However, these epitopes may be potentially validated by *in vitro* neutralization assays to determine the antibody response against ZIKV NS1.

### 3.5 ZIKV E domain-specific conformational B-cell epitopes

**Figure 5:**
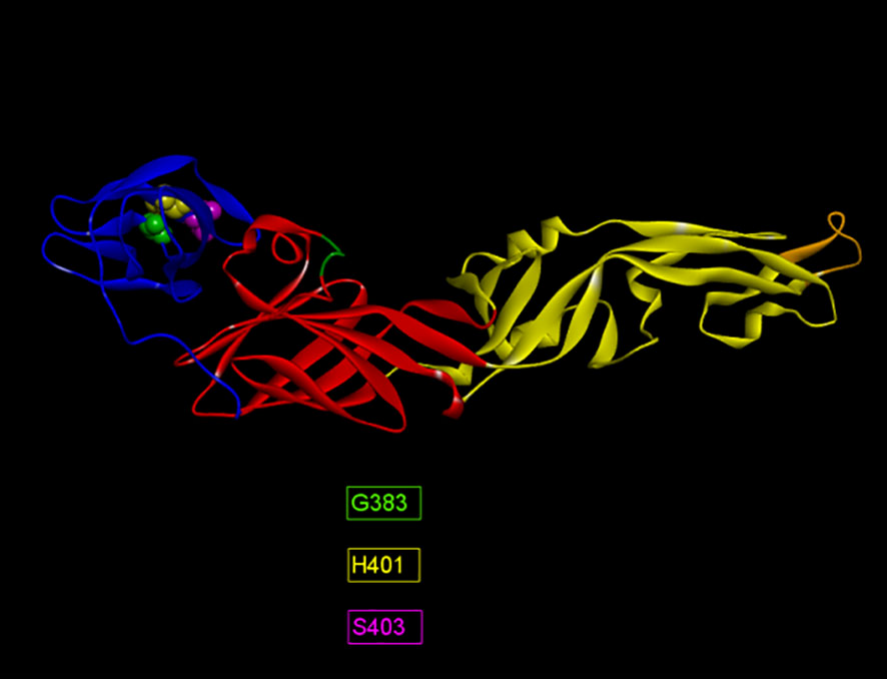
Novel ZIKV E-Specific B-cell conformational epitopes (ZRE).

The humoral immune response to ZIKV infection mostly depends on discontinuous or conformational B-cell epitopes. Alongside linear B-cell epitopes of the ZIKV E, identification of conformational B-cell epitopes is also necessary to evaluate neutralizing antibody responses. The predictions were made by two immunoinformatic tools available at IEDB; Discotope 2.0 and ElliPro. A total of 19 and 14 epitopes were identified by Discotope 2.0 for ZIKV_RAJ and ZIKV_MAH respectively. Ellipro predictions identified 28 and 24 epitopes for ZIKV_RAJ and ZIKV_MAH, respectively. The epitopes common in both prediction tools for ZIKV_RAJ were identified as G383, D384, H401 and S403 (**Supplementary Table S9**). Out of these, D384 was also found in the IEDB list of ZIKV E-specific neutralizing conformational epitopes, whereas the other three epitopes, G383, H401 and S403 were found to be unique, hence considered as novel epitopes for ZIKV_RAJ (**Figure 5**). All these three epitopes were located in EDIII. Similarly, in the case of ZIKV_MAH, epitope W101 of EDI (**Supplementary Table S9**) was found to be the common epitope from both the prediction tools and was also found in the IEDB list of ZIKV E-specific neutralizing conformational epitopes, hence was not considered as novel (**Figure 5**). The presence of different conformational epitopes across the ZIKV E domains and the identification of the above-mentioned novel epitopes is essential in understanding the neutralizing antibody response associated with ZIKV-specific mAbs identifying these epitopes.

### 3.6 ZIKV NS1 domain-specific conformational B-cell epitopes

**Figure 6:**
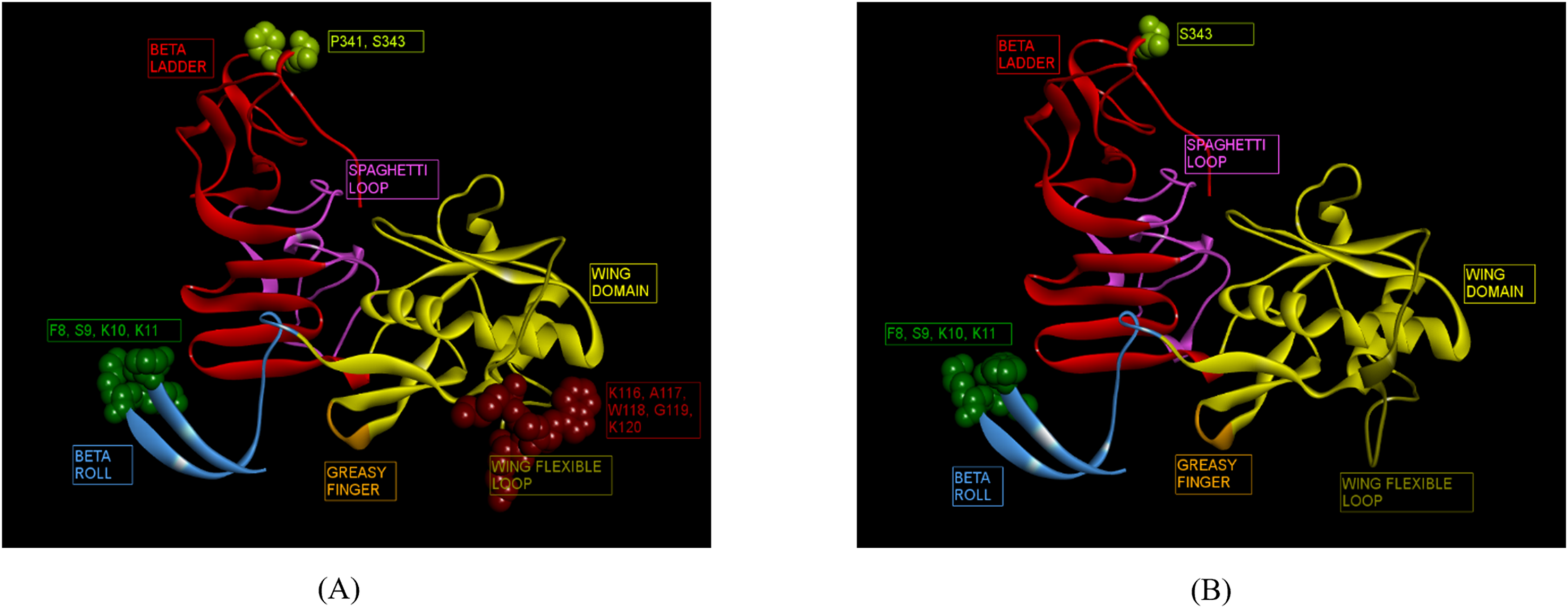
Novel ZIKV NS1 domain-specific B-cell conformational epitopes. **(A)** ZIKV_RAJ NS1 domain-specific B-cell conformational epitopes, **(B)** ZIKV_MAH NS1 domain-specific B cell conformational epitopes.

Immunoinformatics tools Discotope 2.0 and ElliPro were used for the predictions. A total of 37 and 19 conformational epitopes were predicted for ZIKV_RAJ and ZIKV_MAH, respectively with the Discotope 2.0 whereas 75 and 68 epitopes were predicted for ZIKV_RAJ and ZIKV_MAH, respectively by ElliPro. Fourteen epitopes for ZIKV_RAJ and 6 for ZIKV_MAH, common in both prediction tools, were considered for further analysis. (**Supplementary Table S10**). Further comparison of these epitopes with the existing IEDB database of conformational B-cell neutralizing epitopes of ZIKV NS1, revealed the identification of novel epitopes as F8, S9, K10, K11, K116, A117, W118, G119, K120, P341 and S343 specific to ZIKV_RAJ (**Figure 6A**). Moreover, F8, S9, K10, K11, and S343 were considered as novel epitopes specific to ZIKV_MAH (**Figure 6B**).

### 3.7 Molecular docking analysis of ZIKV E-specific novel linear epitopes

In the case of ZIKV E protein, there were 7 novel linear B-cell epitopes and 3 novel conformational B-cell epitopes specific to ZIKV_RAJ. Similarly, there were 7 novel linear B-cell epitopes specific to ZIKV_MAH, however, there were no novel conformational B-cell epitopes specific to ZIKV_MAH. The molecular docking of the ZIKV E protein was carried out with two highly potent neutralizing ZIKV-specific mAbs, ZV-67 and Z3L1. ZV-67 is a mouse mAb that has potent neutralization against both African and Asian ZIKV strains, whereas Z3L1 is a human mAb that has effective neutralization against both African and Asian strains. Moreover, both these mAbs neutralized ZIKV *in vitro* and *in vivo* (21, 41). Molecular docking of the E protein using HDock for Indian ZIKV strains with these mAbs revealed that out of the 7 novel linear B-cell epitopes, 4 epitopes specific to both ZIKV_RAJ and ZIKV_MAH showed intermolecular interactions with the complement determining regions (CDRs) of the mAb ZV-67 (**Table 3**). Of these 4 epitopes, one was located in EDIII, one each belonged to EDI and EDII, and one belonged to the hinge region (DI/DIII:280-295). The epitopes of EDIII and hinge regions had point mutations between both the Indian ZIKV strains whereas the other two epitopes belonging to EDI and EDII were conserved between both ZIKV_RAJ and ZIKV_MAH. However, there was only one novel EDII epitope which interacted with the CDRs of the mAb Z3L1. The inter-molecular bonding interactions between the epitope and CDR region residues were analyzed by 2-D interaction maps.

**Table 3:** H-Dock based molecular docking analysis of ZIKV-specific novel linear epitopes of the E-protein.

Novel Linear Epitopes	CDR Interactions	Indian ZIKV-specificity	Epitope/CDR Interactions	2-D Interaction maps	Docking/Binding scores
323-HGTVTVEVQYA-333A (EDIII) 319-HGTVTVEVQYS-329 (EDIII) 338-PCKVPAQM-345 (EDIII)	Yes Yes No	ZIKV_RAJ (Unique) ZIKV_MAH (Unique) ZIKV_RAJ and ZIKV_MAH	323-HG-324/ CDR3-VH 319-HG-320 CDR3-VH N.A.	323H: 1 C-H bond and 1 pi-donor H-bond 324G: van der Walls 319G: 1 H-bond and 320G: 1 C-H bond N.A.	-306.07 -291.28
5-GVSNRDFVEGMSGGTW-20 (EDI) 32-TVMAQDKPTVDIELVT-47 (EDI)	Yes No	ZIKV_RAJ and ZIKV_MAH ZIKV_RAJ and ZIKV_MAH	10-DFVE-13/ CDR1-VL (ZIKV_RAJ) 7S; 9-RD-10/CDR3-VL (ZIKV_MAH) N.A N.A.	13E: 1 H-bond 12V: 1 C-H bond 10D & 11F: van der Walls 10D: 1 H-bond 7S: 1 C-H bond and 1 pi-donor H-bond, 9R: van der Walls N.A. N.A.	-285.93 -282.31
224-PWHAGADTGTPHWNNKE-240; (EDII) 220-PWHAGADTGTPHWNNKE-236 (EDII)	Yes	ZIKV_RAJ and ZIKV_MAH	235H; 237-NN-238; 239-KE-240/CDR3-VL (ZIKV_RAJ) 233-NN-234; 231H, 235K/CDR3-VL (ZIKV_MAH)	235H: 1 H-bond 237N: 1 H-bond 238N: 1 H-bond 239K: van der Walls 233N: 1 H-bond 234N: 1 H-bond 231H: van der Walls 235K: van der Walls	-276.14 -277.38
43-IELVTTTVSNMAEVRS-58 (Hinge Region) 280-TKGRLSSGHLKCRLKMDK-297 (Hinge Region) 276-AKGRLSSGHLKCRLKMDK-293 (Hinge Region)	No Yes Yes	ZIKV_RAJ and ZIKV_MAH ZIKV_RAJ ZIKV_MAH	N.A 288H;290K/CDR1-VL 284H/CDR3-VH	N.A 288H: van der Walls 290K: van der Walls 284H: van der Walls	-286.01 -283.06

Molecular Docking interactions of the novel epitopes with potent mouse monoclonal antibody ZV67; N.A. (Not Available).

Amino acid mutations are coloured in Red with respect to ZIKV Prototype strain MR766 (Green).

**Figure 7:**
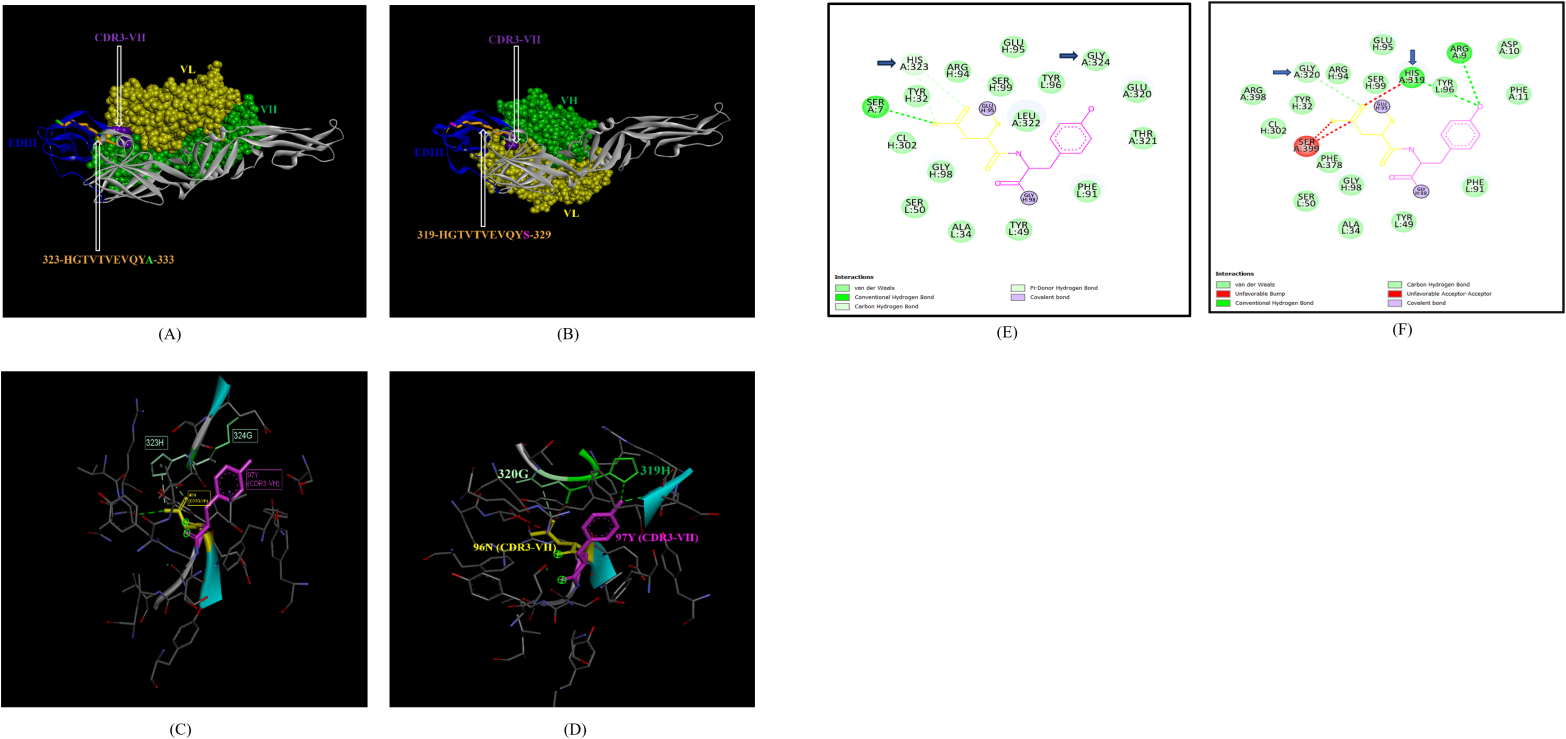
Molecular docking of novel linear EDIII epitopes with CDRs of ZV-67 mAb. **(A)** 3-D model of docked ZRE and ZV67; VL (coloured in yellow), VH (coloured in light green), CDR3-VH (coloured in purple) interacting with EDIII (coloured in blue) epitope 323-HGTVTVEVQYA-333 (coloured in light brown) except alanine (coloured in light green). **(B)** 3-D model of docked ZME and ZV67; VL (coloured in yellow), VH (coloured in light green), CDR3-VH (coloured in purple) interacting with EDIII (coloured in blue) epitope 319-HGTVTVEVQYS (coloured in teal orange) except serine (coloured in blue). **(C)** Epitope-CDR interaction in 3-D for ZRE with CDR3-VH of ZV67; 323-HG-324 (coloured in shades of light green) are the amino acids of EDIII epitope interacting with CDR3-VH amino acid residues, 96-NY-97 (coloured in pink and yellow, respectively). **(D)** Epitope-CDR interaction in 3-D for ZME with CDR3-VH of ZV67; 319-HG-320 (coloured in shades of light green) are the amino acids of EDIII epitope interacting with CDR3-VH amino acid residues, 96-NY-97 (coloured in pink and yellow, respectively). **(E)** 2-D interaction map of panel **(C)**; blue arrows indicating 323H forming one carbon-hydrogen and one pi-donor hydrogen bond and 324G forming van der Walls interactions with 96N and 97Y. **(F)** 2-D interaction map of panel **(D)**; blue arrows indicating 319H forming conventional hydrogen bond and 320G forming carbon-hydrogen bond with 96N and 97Y.

In the case of EDIII, epitope 323-HGTVTVEVQYA-333, unique to ZIKV_RAJ (**Figure 7A**) and epitope 319-HGTVTVEVQYS-329 unique to ZIKV_MAH (Readers should note that due to a deletion of 4-amino acids in ZIKV_MAH strain at N154, the nomenclature is: amino acid 319 for ZIKV_MAH is equivalent to 323 for ZIKV_RAJ which applies to all the amino acids post 154^th^ position in ZIKV_MAH) (**Figure 7B**), the epitope residues 323-HG-324 (ZIKV_RAJ; **Figure 7C**) and 319-HG-323 (ZIKV_MAH; **Figure 7D**) interacted with CDRs of VH and VL regions of mAb ZV-67. The 2-D interaction maps for these epitopes revealed intermolecular hydrogen bonding interactions with CDRs (**Table 3**) of ZV-67, wherein the epitope-CDR3(VH) interactions were found to be the most suitable. In the case of the epitope unique to ZIKV_RAJ (323-HGTVTVEVQYA-333), the 2-D interaction maps showed that 323H formed one carbon-hydrogen (C-H) bond and one pi-donor hydrogen bond and 324G formed van der Walls interaction with CDR3-VH residues, respectively (**Figure 7E**). However, in the case of the epitope unique to ZIKV_MAH (319-HGTVTVEVQYS-329), the results were slightly different with 319H forming one conventional hydrogen (H) bond and 320G forming one carbon-hydrogen (C-H) bond with CDR3-VH of ZV-67, respectively (**Figure 7F**). The mAb ZV-67 is known to bind to the ZIKV-E protein in the EDIII domain (21).

**Figure 8:**
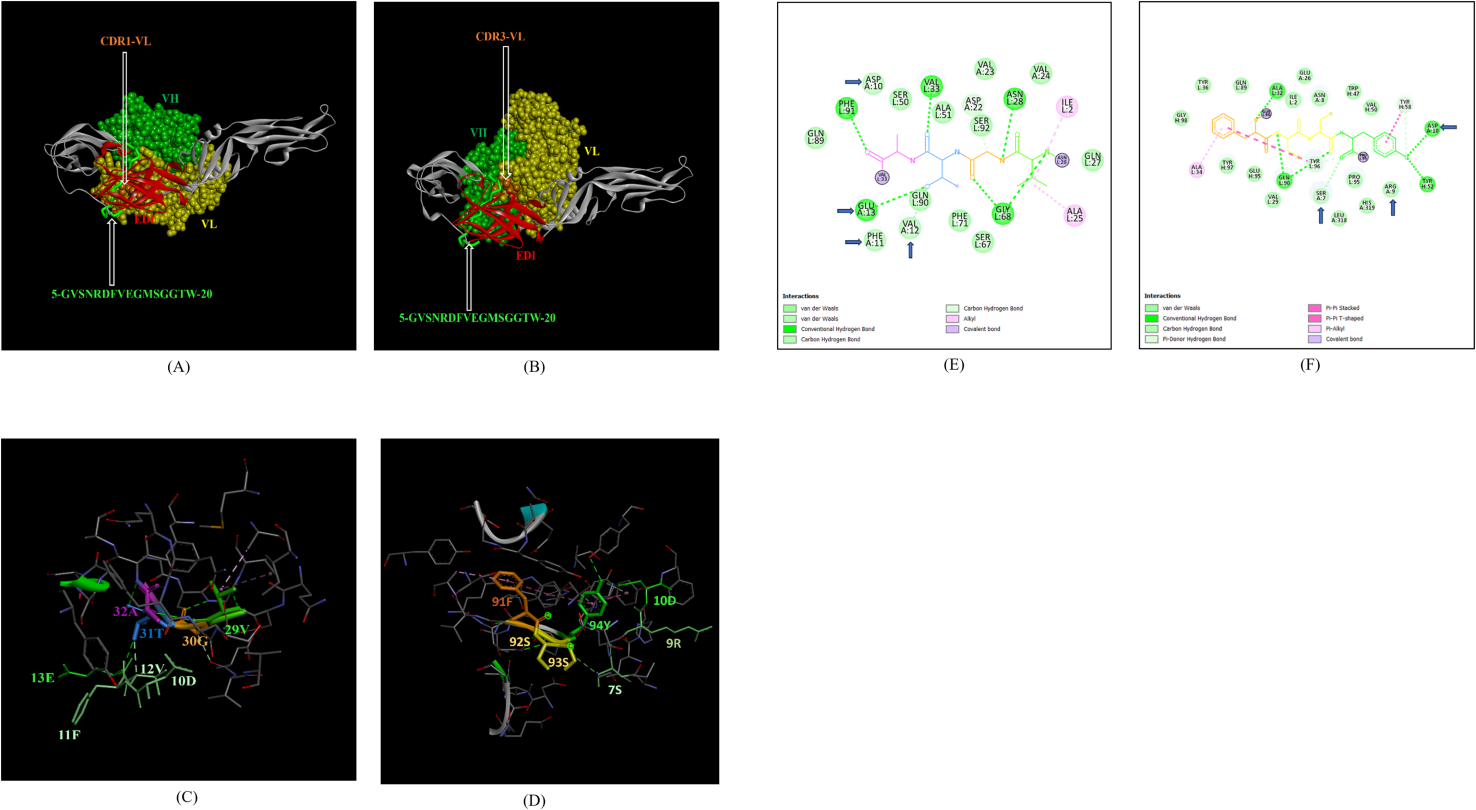
Molecular docking of novel linear EDI epitopes with CDRs of ZV-67 mAb: **(A)** 3-D model of docked ZRE and ZV67; VL (coloured in yellow), VH (coloured in light green), CDR-1-VL (coloured in dark orange) interacting with EDI (coloured in red) epitope 5-GVSNRDFVEGMSGGTW-20 (coloured in light green). **(B)** 3-D model of docked ZME and ZV67; VL (coloured in yellow), VH (coloured in light green), CDR3-VL (coloured in dark orange) interacting with EDI (coloured in red) epitope 5-GVSNRDFVEGMSGGTW-0 (coloured in light green). **(C)** Epitope-CDR interaction in 3-D for ZRE with CDR1-VL of ZV67; 10-DFVE-13 (coloured in shades of light green) are the amino acids of EDI epitope interacting with CDR1-VH amino acid residues, 29-VGTA-31 (coloured in light green, orange, purple and light blue, respectively). **(D)** Epitope-CDR interaction in 3-D for ZME with CDR3-VL of ZV67; 7S, 9-RD-10 (coloured in shades of light green) are the amino acids of EDI epitope interacting with CDR3-VL amino acid residues, 91-FSSY (coloured in dark orange, yellow, yellow and light green, respectively). **(E)** 2-D interaction map of panel **(C)**; blue arrows indicating 13E forming one conventional hydrogen bond, 12E forming one carbon-hydrogen bond and 10-DF-11 forming van der Walls interactions with 31T, 32A, 30G and 29Y, respectively. **(F)** 2-D interaction map of panel **(D)**; blue arrows indicating 10D forming one conventional hydrogen bond, 7S forming one carbon-hydrogen bond, and 9R forming van der Walls interactions with 94Y, 93S,92S and 91Y.

**Figure 9:**
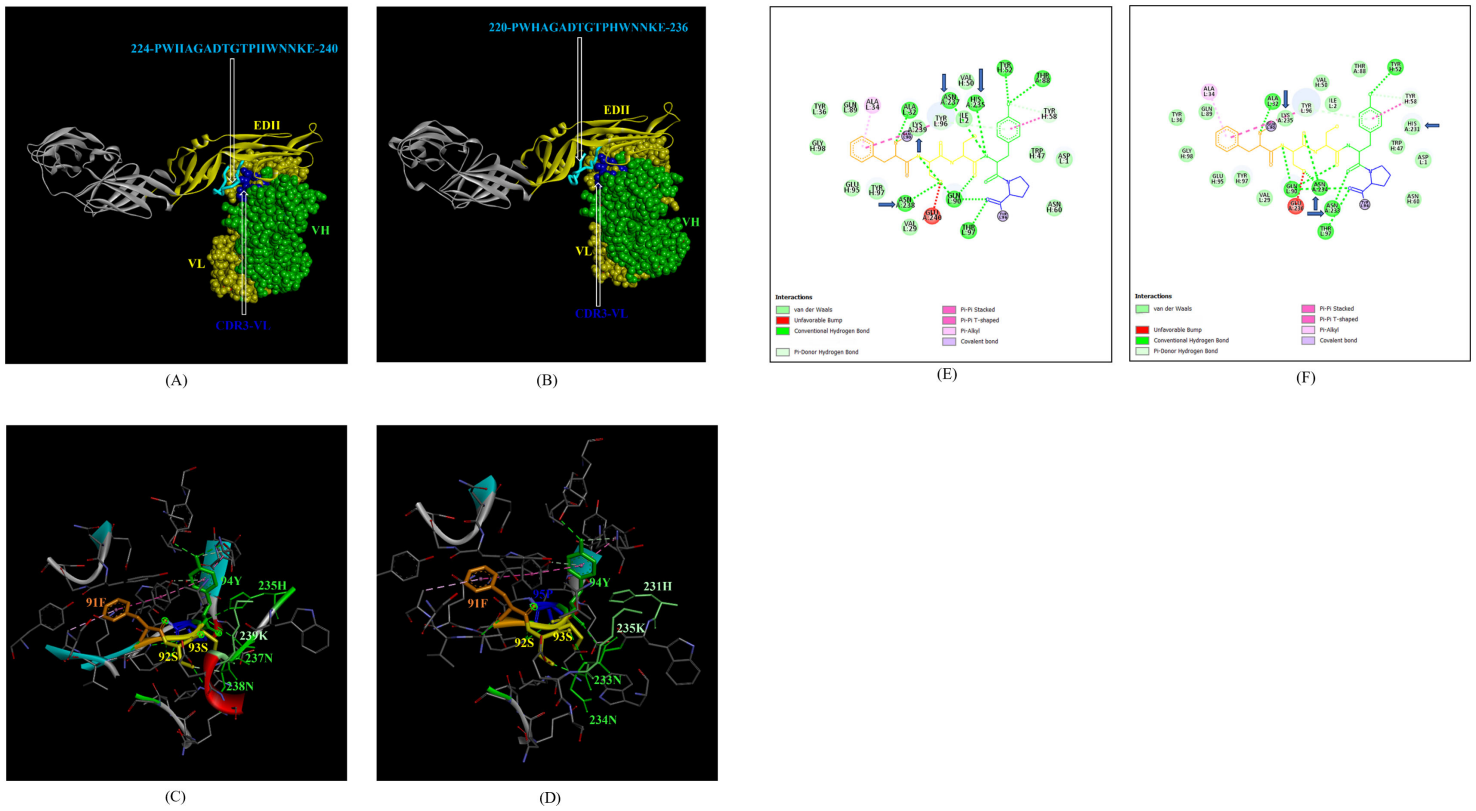
Molecular docking of novel linear EDII epitopes with CDRs of ZV-67 mAb. **(A)** 3-D model of docked ZRE and ZV67; VL (coloured in yellow), VH (coloured in light green), CDR3-VL (coloured in blue) interacting with EDII (coloured in yellow) epitope 224-PWHAGADTGTPHWNNKE-240 (coloured in light blue). **(B)** 3-D model of docked ZME and ZV67; VL (coloured in yellow), VH (coloured in light green), CDR3-VL (coloured in blue) interacting with EDII (coloured in yellow) epitope 224-PWHAGADTGTPHWNNKE-240 (coloured in light blue). **(C)** Epitope-CDR interaction in 3-D for ZRE with CDR3-VL of ZV67; 235H, 237-NNK-239 (coloured in shades of light green) are the amino acids of EDI epitope interacting with CDR3-VH amino acid residues, 91-FSSYP-95 (coloured in dark orange, yellow, yellow, light green and blue, respectively). **(D)** Epitope-CDR interaction in 3-D for ZME with CDR3-VL of ZV67; 235H, 237-NNK-239 (coloured in shades of light green) are the amino acids of EDI epitope interacting with CDR3-VH amino acid residues, 91-FSSYP-95 (coloured in dark orange, yellow, yellow, light green and blue, respectively). **(E)** 2-D interaction map of panel **(C)**; blue arrows indicating 235H forming one conventional hydrogen bond, 237-NN-238 forming one carbon-hydrogen bond each and 239K forming van der Walls interaction with 91F, 92S, 93S, 94F and 95P. **(F)** 2-D interaction map of panel **(D)**; blue arrows indicating 233-NN-234 forming one carbon-hydrogen bond each and 231H and 235K forming van der Walls interaction with 91F, 92S, 93S, 94F and 95P.

**Figure 10:**
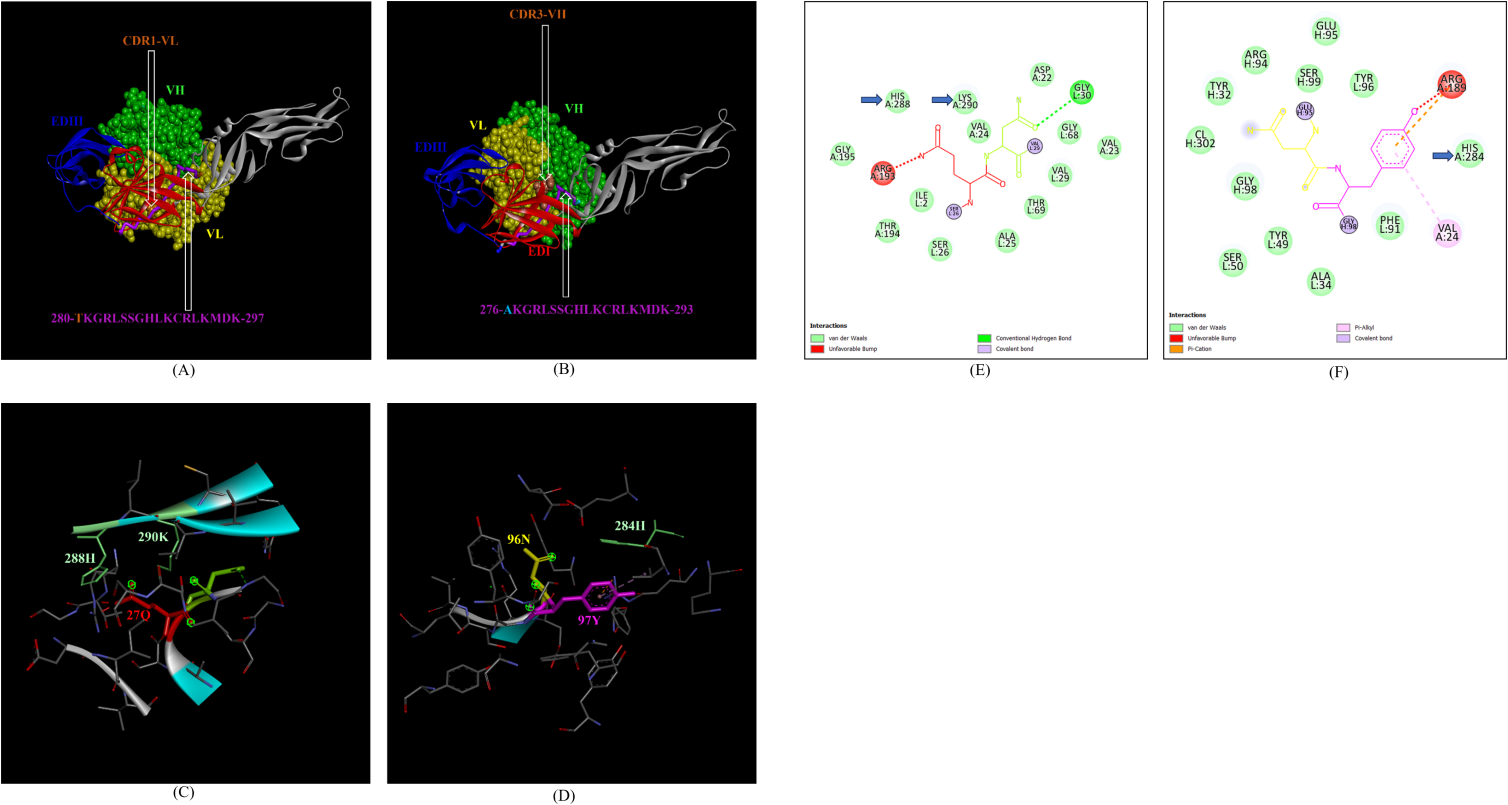
Molecular docking of novel linear hinge region epitopes with CDRs of ZV-67 mAb. **(A)** 3-D model of docked ZRE and ZV67; VL (coloured in yellow), VH (coloured in light green), CDR1-VL (coloured in brown) interacting with DI/DIII hinge region (coloured in red/blue) epitope 280-TKGRLSSGHLKCRLKMDK-297 (coloured in purple; except Threonine which is colored in brown). **(B)** 3-D model of docked ZME and ZV67; VL (coloured in yellow), VH (coloured in light green), CDR3-VH (coloured in brown) interacting with DI/DIII hinge region (coloured in red/blue) epitope 276-AKGRLSSGHLKCRLKMDK-293 (coloured in purple; except alanine which is colored in light brown). **(C)** Epitope-CDR interaction in 3-D for ZRE with CDR1-VL of ZV67; 288H and 290K (coloured in shades of light green) are the amino acids of DI/DIII hinge region epitope interacting with CDR1-VL amino acid residues, 27-QN-28 (coloured in red and green, respectively). **(D)** Epitope-CDR interaction in 3-D for ZME with CDR3-VH of ZV67; 284H (coloured in shades of light green) is the amino acid of DI/DIII hinge region epitope interacting with CDR3-VH amino acid residues, 96-NY-97 (coloured in yellow and purple, respectively). **(E)** 2-D interaction map of panel **(C)**; blue arrows indicating 288H and 290K forming with van der Walls interaction with 27Q and 28N. **(F)** 2-D interaction map of panel **(D)**; blue arrows indicating 284H forming van der Walls interaction with 96N and 97Y.

In addition, the footprint of mAb ZV-67 also covered parts of EDI and EDII domains and interactions with novel epitopes were observed. Out of the two novel EDI epitopes, epitope 5-GVSNRDFVEGMSGGTW-20 interacted with CDR regions of the mAb ZV-67. This epitope was conserved in both the Indian ZIKV strains. The most suitable docking interactions shown by this EDI epitope were with CDR1-VL in the case of ZIKV_RAJ and CDR3-VL in the case of ZIKV_MAH, respectively (**Figures 8A–D**). The 2-D interaction maps showed that for the ZIKV_RAJ EDI, the amino acids 13E and 12V showed one conventional hydrogen (H)-bonding interaction and one carbon-hydrogen (C-H) bonding interaction, respectively with CDR1-VL residues (**Figure 8E**) Amino acids 10D and 11F displayed van der Walls interactions (**Figure 8E**). However, for ZIKV_MAH EDI, amino acids 10D showed one conventional hydrogen (H)-bond, and 7S displayed one carbon-hydrogen (C-H) bond and one pi-donor hydrogen bond with CDR3-VL residues (**Figure 8F**). In this case, amino acid 9R showed van der Walls interactions (**Figure 8F**). Further, the novel EDII epitope 224-PWHAGADTGTPHWNNKE-240 which was conserved in both ZIKV_RAJ and ZIKV_MAH, interacted with CDR3-VL of the mAb (**Figures 9A–D**). For ZIKV_RAJ EDII, the amino acids 235H, 237N, and 238N had 1 conventional H-bond each and amino acids 239K and 240E had van der Walls interaction. (**Figure 9E**). However, in the case of ZIKV_MAH EDII (220-PWHAGADTGTPHWNNKE-236) the amino acids 233N and 234N had 1 conventional H-bond each, with 234N also displaying an unfavorable interaction. Further, 231H and 235K possessed van der Walls interaction (**Figure 9F**). There were two novel epitopes identified in the hinge regions of Indian ZIKV strains, among which the epitope 280-TKGRLSSGHLKCRLKMDK-297 (ZIKV_RAJ) and epitope 276-AKGRLSSGHLKCRLKMDK-293 (ZIKV_MAH) had interactions with the CDRs of ZV-67, respectively (**Figures 10A–D**) The 2-D interaction maps of 280-TKGRLSSGHLKCRLKMDK-297 showed that amino acids 288H and 290K had van der Walls interaction with CDR1-VL of the mAb (**Figure 10E**); whereas epitope 276-AKGRLSSGHLKCRLKMDK-293 had only 284H having van der Walls interaction with CDR3-VL (**Figure 10F**).

**Figure 11:**
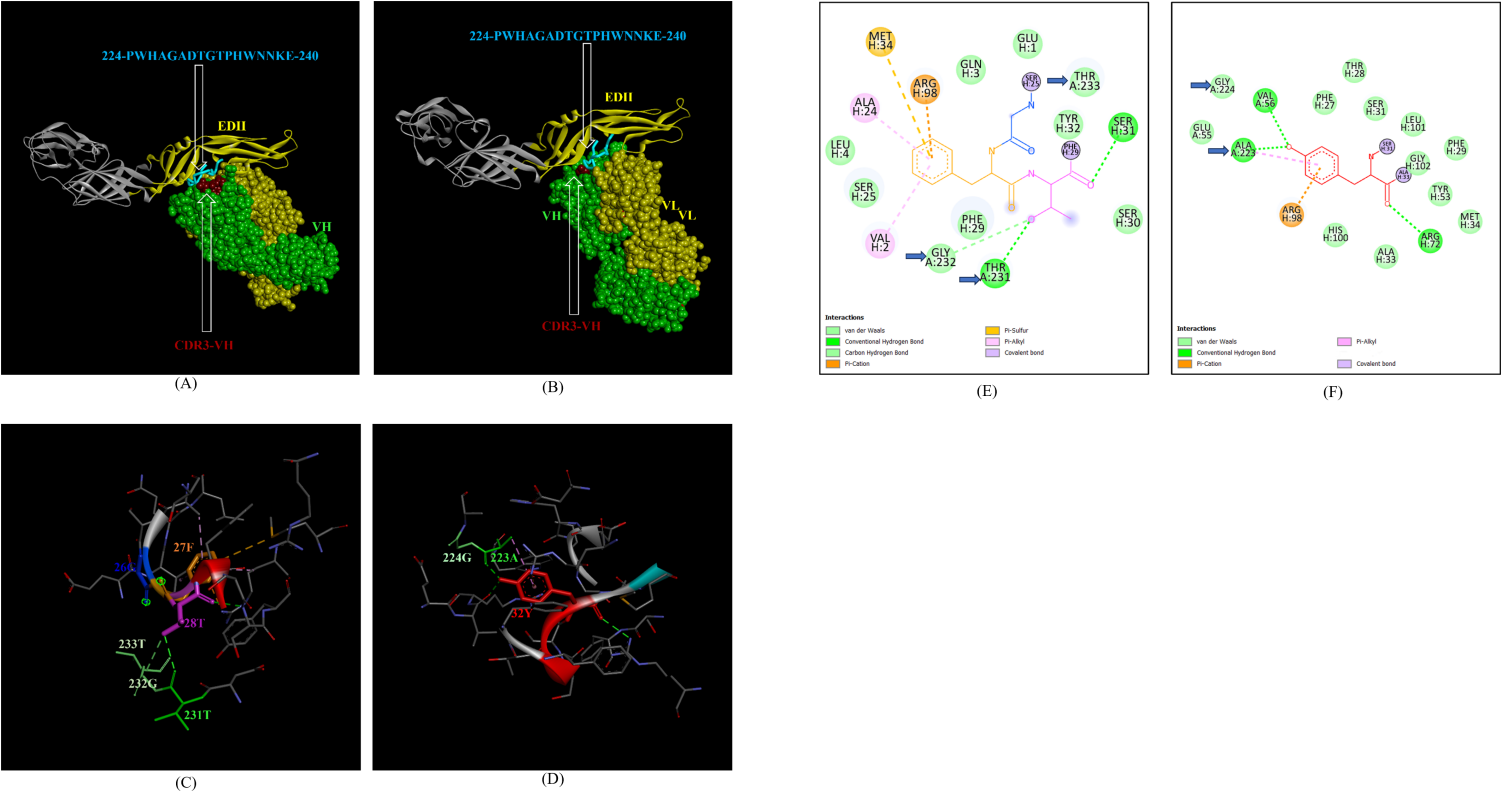
Molecular docking of novel linear EDII epitopes with CDRs of Z3L1 mAb. **(A)** 3-D model for docked ZRE and Z3L1; VL (coloured in yellow), VH (coloured in light green), CDR1-VH (coloured in magenta) interacting with EDII (coloured in yellow) epitope 224-PWHAGADTGTPHWNNKE-240 (coloured in light blue). **(B)** 3-D model for docked ZME and Z3L1; VL (coloured in yellow), VH (coloured in light green), CDR1-VH (coloured in magenta) interacting with EDII (coloured in yellow) epitope 224-PWHAGADTGTPHWNNKE-240 (coloured in light blue). **(C)** Epitope-CDR interaction in 3-D for ZRE with CDR1-VH of Z3L1; 231-TGT-233 colored in shades of light green) are the amino acids of EDII epitope interacting with CDR1-VL amino acid residues 26-GFT-28 (coloured in blue, orange and purple, respectively). **(D)** Epitope-CDR interaction in 3-D for ZME with CDR1-VH of Z3L1; 223-AG-224 (coloured in shades of light green) are the amino acids of EDII epitope interacting with CDR1-VL amino acid residue 32Y (coloured in red). **(E)** 2-D interaction map of panel **(C)**; blue arrows indicating 231T forming one conventional hydrogen bond, 232G forming one carbon-hydrogen bond and 233T forming van der Walls interaction with 26G, 27F, and 28T. **(F)** 2-D interaction map of panel **(D)**; blue arrows indicating 223A forming one conventional hydrogen bond and one pi-alkyl bond, 224G forming van der Walls interaction with 32Y. arrows indicating 223A (forming one H bond and one pi-alkyl bond), and 224G (forming van der Walls interaction) with 32Y.

Next, we sought to identify the epitope-CDR interaction of the Indian ZIKV strains with human mAb Z3L1. The novel epitope of EDII 224-PWHAGADTGTPHWNNKE-240, conserved in both ZIKV_RAJ and ZIKV_MAH, was the only epitope showing molecular interactions with the CDRs (**Table 4**) of Z3L1. However, novel epitopes in other E-domains also interacted with Z3L1, but these interactions were not with CDR regions. On the other hand, this EDII epitope interacted with CDR1-VH of Z3L1 in the case of both the Indian ZIKV strains (**Table 4**; **Figures 11A, B**). This displayed different epitope-CDR bonding interactions specific for ZIKV_RAJ (**Figures 11A, C**) and ZIKV_MAH (**Figures 11B, D**) which were demonstrated by their 2-D interaction maps. The epitope 224-PWHAGADTGTPHWNNKE-240 had amino acids 231T, 232G and 233A forming 1 H-bond, 1 C-H bond and van der Walls interaction, respectively which were specific to ZIKV_RAJ (**Figure 11E**) whereas the same epitope (220-PWHAGADTGTPHWNNKE-236) had amino acids 223A forming 1 H-bond and 1 pi-alkyl bond and 224G forming van der Walls interaction in the case of ZIKV_MAH (**Figure 11F**)

**Table 4:** H-Docked based molecular docking analysis of ZIKV-specific Novel Linear Epitopes of the E-protein.

Novel Linear Epitopes	CDR Interactions	Indian ZIKV-specificity	Epitope/CDR Interactions	2-D Interaction maps	Docking/Binding scores
323-HGTVTVEVQYA-333 (EDIII) 319-HGTVTVEVQYS-329 (EDIII) 338-PCKVPAQM-345 (EDIII)	No No No	ZIKV_RAJ (Unique) ZIKV_MAH (Unique) ZIKV_RAJ and ZIKV_MAH	N.A. N.A N.A.	N.A. N.A. N.A.	N.A. N.A. N.A.
5-GVSNRDFVEGMSGGTW-20 (EDI) 32-TVMAQDKPTVDIELVT-47 (EDI)	No No	ZIKV_RAJ and ZIKV_MAH ZIKV_RAJ and ZIKV_MAH	N.A N.A.	N.A. N.A.	N.A. N.A.
224-PWHAGADTGTPHWNNKE-240 (EDII); 220-PWHAGADTGTPHWNNKE-236 (EDII)	Yes	ZIKV_RAJ and ZIKV_MAH	231-TGA-233/CDR1-VH (ZIKV_RAJ) 223-AG-24/CDR1-VH (ZIKV_MAH)	231T: 1 H-bond 232G: 1 C-H bond 233A: van der Walls 223A: 1 H-bond and 1 pi-alklyl bond 224G: van der Walls	-253.17 -242.52
43-IELVTTTVSNMAEVRS-58 (Hinge) 280-TKGRLSSGHLKCRLKMDK-297 (Hinge) 276-AKGRLSSGHLKCRLKMDK-293 (Hinge)	No No No	ZIKV_RAJ and ZIKV_MAH ZIKV_RAJ (Unique) ZIKV_MAH (Unique)	N.A N.A N.A	N.A N.A N.A	N.A. N.A. N.A.

Molecular Docking interactions of the novel epitopes with potent human monoclonal antibody Z3L1; N.A. (Not Available).

Amino acid mutations are coloured in Red with respect to ZIKV Prototype strain MR766 (Green).

## 4 Discussion

In the case of emerging Flavivirus infection, such as the Zika virus, E and NS1 proteins play a vital role in eliciting robust humoral immunity (19, 26). The ZIKV-specific B-cell epitopes are crucial in inducing the humoral immune response (15, 42, 43). The advent of immunoinformatics has led to the prediction of various B-cell epitopes in Flaviviruses’ antigens. However, a similar broad-spectrum analysis of the ZIKV-specific B-cell epitopes is required and remains poorly understood. In-silico prediction and validation of these epitopes may help in ZIKV therapeutics and vaccine design.

In this study, we identified B cell epitopes of E and NS1 proteins of two co-circulating Indian ZIKV strains. Further, these epitopes were mapped to their modelled 3-D structures leading to the identification of novel epitopes based on the stringent study design criteria. Molecular docking with potent ZIKV-neutralizing mAbs validated our findings via epitope-CDR interactions. The identification of these novel epitopes was specific and unique to Indian ZIKV strains. These epitopes have the potential to induce peptide-specific antibodies, mostly involved in neutralizing response, especially against the ZIKV E protein.

In-silico antigenic characterization of viral proteins is essential for epitope identification. The E protein of ZIKV plays an important role in virus entry, attachment, and fusion. Apart from these functions, the ZIKV E protein is the major target of neutralizing antibodies. It consists of three domains: the central beta-barrel domain (EDI), an elongated finger-like domain (EDII) consisting of a hydrophobic fusion loop (FL) and an IgC-like immunoglobulin domain (EDIII) (21, 26, 40). Moreover, these domains display differential neutralizing potential, with EDIII being the most potent and ZIKV-type specific. Earlier studies reported antigenicity analyses for the whole E protein of the Zika virus (40). However, our study involves domain-wise estimation of antigenicity for ZIKV E protein for different strains, which provides detailed insight into understanding the antigenic characteristics: similarities and differences between the strains. Following domain-specific antigenicity analysis, we predicted linear and conformational B-cell epitopes specific to ZIKV E protein as the overall humoral immune response depends on both the primary and tertiary structures of these epitopes (44, 45). The prediction of the linear and conformational epitopes was carried out using a combination of immunoinformatic tools to minimize false positive results and the amino acid regions predicted as epitopes by all the different tools were considered for further analyses (27, 33, 46). Moreover, the length of linear B-cell epitopes was also considered as it is a significant parameter in the case of designing peptide vaccines capable of inducing substantial humoral immune response (47). The experimentally validated ZIKV B-cell neutralizing epitopes mostly range from 14-22 residues which overlaps with our predicted epitopes. The novel epitopes that displayed epitope-CDR interactions range from 11-18 residues in length which makes them good candidates to be incorporated into peptide vaccines. Apart from considering the antigenicity of the shortlisted epitopes, their non-allergenicity and non-toxicity were also examined which are important factors to consider in the case of designing multi-epitope proteins (12).

Besides epitope mapping and visualization, three-dimensional models of the proteins are essential to study epitope recognition and accessibility by the antibodies (48). The template identified for ZIKV_RAJ was 7YW8.pdb whereas the template for ZIKV_MAH E protein identified as 7YW7.pdb had a deletion of 4 amino acids in the E-glycan loop (N154-157) (49). ZIKV strains with E glycan loop deletions, especially in Asia, need to be studied extensively to understand their effect on virus infectivity, immunity, and pathogenesis. The predicted structure’s precision and stoichiometry were determined by the Ramachandran plot, which identified sterically allowed and disallowed regions (50). The most suitable 3-D models had zero per cent amino acids in disallowed regions and around >99% in the favorably allowed regions. Based on our study design we could identify novel linear and conformational epitopes for ZIKV E protein.

The stringent study criteria for selecting the novel epitopes were further validated *in silico* by studying their interactions with CDRs of the highly neutralizing ZIKV E-protein specific mouse and human mAbs. Interaction of the epitopes with the CDR regions of the antibody leads to enhanced binding affinity resulting in specific neutralizing response (51). Considering this, we hypothesized to select the CDRs of two highly neutralizing mAbs ZV-67 and Z3L1 to carry out the docking with ZIKV E protein. These two mAbs were selected as they were specific to ZIKV E protein neutralization, possessed high resolution X-ray crystallographic structures, and were effective across both African and Asian ZIKV strains *in vitro* and *in vivo* (21, 41). We identified that the novel linear epitopes across all three domains and DI-DIII hinge regions interacted with CDRs of ZV-67 mAb, which highlights the fact that the Indian ZIKV strains had broad-spectrum epitope-CDR interactions with this mouse mAb (**Table 3**). However, in the case of the human mAb Z3L1, only the novel epitope of EDII showed CDR interactions (**Table 4**). 3-D and 2-D interaction maps are essential to study the different bonds that form during epitope-CDR binding. All the novel epitopes, being identical or having point mutations between the ZIKV_RAJ and ZIKV_MAH displayed differences in their 3-D and 2-D receptor-ligand interaction maps, suggesting strain-specific differences in forming bonds with the mAbs’ CDRs.

Our predictions also identified the EDIII epitopes 363-PVITESTENSK-373 (ZIKV_RAJ) and 363-PVITESAENSK-373 (ZIKV_MAH), which overlapped with critical residues involved in neutralization by the mAbs ZV-2 and ZV-67 (21). Similarly, epitopes 384-DSYIVIGVGDKKITHHWHRS-403 and 384-DSYIVIGVGEKKITHHWHRS-403 overlapped with key residues required for neutralization with mAbs ZV-48, ZV-64 and ZV-67 (21, 52). As, our prediction spanned across all three domains, including hinge regions, EDII epitope 61-YEASISDMASDSRCPT-76, and DI-DII hinge epitope 118-KFACSKKMTGSIQPE-133 (ZIKV_RAJ and ZIKV_MAH) were also part of key residues required for neutralization by mAb ZIKV-117 (53). Moreover, the mAb Z3L1 also had neutralizing epitopes overlapping with our predicted epitope 126-TGKSIQPENLEYRIMLSV-143 in the DI-DII hinge (41).

The ZIKV NS1 protein is another major target of neutralizing antibodies, following the immunodominant ZIKV E protein (23–26, 54–56). Hence, immunoinformatic analyses of ZIKV NS1 were undertaken. The ZIKV NS1 has three distinct domains: the beta roll (residues 1-29), the wing domain (residues 30-180) and the beta ladder (residues 181-352). Connector residues (33, 35–38, 41, 57, 58) and (152–180) within the wing domain link it to the beta roll and beta-ladder domain, respectively (54). Domain-wise antigenicity analysis revealed the beta-roll and beta ladder domains to be antigenic while the wing domain to be non-antigenic. The non-antigenic values of the wing domain may be attributed to the connector residues which form a part of the inner hydrophobic surface of NS1 (59). However, barring these residues, the wing domain is targeted by ZIKV NS1-specific mAbs that confer protection (24). These observations conveyed the importance of the wing domain in ZIKV protection and hence it was considered for epitope identification, alongside the antigenic beta roll and beta ladder domains.

Both ZIKV_RAJ and ZIKV_MAH NS1 had identical templates i.e. 5K6K. pdb (25) for 3-D structure predictions. As there was unavailability of linear ZIKV NS1-specific neutralizing epitopes at IEDB, we were not able to ascertain the novel linear epitopes, but there was concordance of our predicted epitopes with critical residues of ZIKV NS1 neutralizing mAbs, such as 3G2, 4B8, 2E11, 14G5, AA12, EB9 and GB5 (54, 55, 60, 61). However, there were two epitopes 10-KKETRCGTGVFVYNDVE-26 (beta roll) and 83-GIQLTVVVGSVKNP-96 (wing domain) found exclusively in both Indian ZIKV strains which did not overlap with the critical residues of known ZIKV NS1 mAbs, suggesting that these may be important to study as novel ZIKV NS1 epitopes. Among them, epitope 83-GIQLTVVVGSVKNP-96 did not belong to the hydrophobic connector residues of the wing domain, therefore it may be important in studying ZIKV NS1 protection. However, in the case of ZIKV NS1, epitope-CDR interactions were not possible to analyze due to the unavailability of resolved X-ray crystallographic structures of ZIKV NS1 neutralizing mAbs.

## 5 Conclusion

To summarize, we conducted detailed antigenic characterization of the E and NS1 proteins for co-circulating Indian strains of ZIKV with domain-specific analyses. This helped in the identification of novel epitopes in E and NS1 proteins having zero percent identity with the amino acid compositions of previously reported ZIKV-neutralizing epitopes. Molecular docking studies further revealed that some of the novel epitopes of E protein are being recognized by known ZIKV-neutralizing antibodies. Our studies on *in vitro* and *in vivo* experiments targeting these novel epitopes to understand the key role in humoral immunity are in progress. Therefore, the findings will help in the development of multi-epitope proteins for diagnostics and vaccinology applications in future.

## Data Availability

The original contributions presented in the study are included in the article/**Supplementary Material**, further inquiries can be directed to the corresponding author/s.

## Supplementary material

Supplementary Figure 1 Multiple sequence alignment of E protein in ZIKV strains: ZIKV MR766 (coloured in green), ZIKV NATAL RGN (coloured in red), ZIKV_RAJ (coloured in blue), and ZIKV_MAH (coloured in purple). All the mutations are highlighted in yellow with respect to ZIKV MR766.

Supplementary Figure 2 Multiple sequence alignment of NS1 protein in ZIKV strains; ZIKV MR766 (coloured in green), ZIKV NATAL RGN (coloured in red), ZIKV_RAJ (coloured in blue), and ZIKV_MAH (coloured in purple). All the mutations are highlighted in yellow with respect to ZIKV MR766.

Supplementary Figure 3 3-D Templates for Indian ZIKV E; **(A)** 7YW8 (ZIKV_RAJ), **(B)** 7YW7 (ZIKV_MAH) where Chain A is highlighted in yellow.

Supplementary Figure 4 3-D Template for Indian ZIKV NS1: 5K6K (ZIKV_RAJ and ZIKV_MAH) where Chain A is highlighted in yellow.

Supplementary Figure 5 Graphical representation of linear B-cell epitopes by BepiPred 2.0 method (yellow peaks = predicted epitopes, and green inverted peaks = non-epitopes) for both Indian ZIKV E (Threshold=0.5). **(A)** ZIKV_RAJ and **(B)** ZIKV_MAH.

Supplementary Figure 6 Graphical representation of linear B-cell epitopes by Kolaskar and Tongaonkar method (yellow peaks = predicted epitopes, and green inverted peaks = non-epitopes) for both Indian ZIKV E. **(A)** ZIKV_RAJ (Threshold=1.026) and **(B)** ZIKV_MAH (Threshold=1.028).

Supplementary Figure 7 Graphical representation of linear B-cell epitopes by Emini surface accessibility method (yellow peaks = predicted epitopes, and green inverted peaks = non-epitopes) for both Indian ZIKV E (Threshold=1.00). **(A)** ZIKV_RAJ and **(B)** ZIKV_MAH.

Supplementary Figure 8 Graphical representation of linear B-cell epitopes by Parker hydrophilicity method (yellow peaks = predicted epitopes, and green inverted peaks = non-epitopes) for both Indian ZIKV E. **(A)** ZIKV_RAJ (Threshold=1.475) and **(B)** ZIKV_MAH (Threshold=1.470).

Supplementary Figure 9 Graphical representation of linear B-cell epitopes by BepiPred 2.0 method (yellow peaks = predicted epitopes, and green inverted peaks = non-epitopes) for both Indian ZIKV NS1 (Threshold=0.5). **(A)** ZIKV_RAJ and **(B)** ZIKV_MAH.

Supplementary Figure 10 Graphical representation of linear B-cell epitopes by Kolaskar and Tongaonkar method (yellow peaks = predicted epitopes, and green inverted peaks = non-epitopes) for both Indian ZIKV NS1. **(A)** ZIKV_RAJ (Threshold=1.018) and **(B)** ZIKV_MAH (Threshold=1.023).

Supplementary Figure 11 Graphical representation of linear B-cell epitopes by Emini surface accessibility method (yellow peaks = predicted epitopes, and green inverted peaks = non-epitopes) for both Indian ZIKV NS1 (Threshold=1.00). **(A)** ZIKV_RAJ and **(B)** ZIKV_MAH.

Supplementary Figure 12 Graphical representation of linear B-cell epitopes by Parker hydrophilicity method (yellow peaks = predicted epitopes, and green inverted peaks = non-epitopes) for both Indian ZIKV NS1. **(A)** ZIKV_RAJ (Threshold=1.701) and **(B)** ZIKV_MAH (Threshold=1.726).
