# Supplementary figures and images for: Identification of B-cell epitopes of Indian Zika virus strains using immunoinformatics
Source: Front Immunol. 2025 Feb 27;16:1534737. doi: 10.3389/fimmu.2025.1534737 (PMC11903408; doi:10.3389/fimmu.2025.1534737)

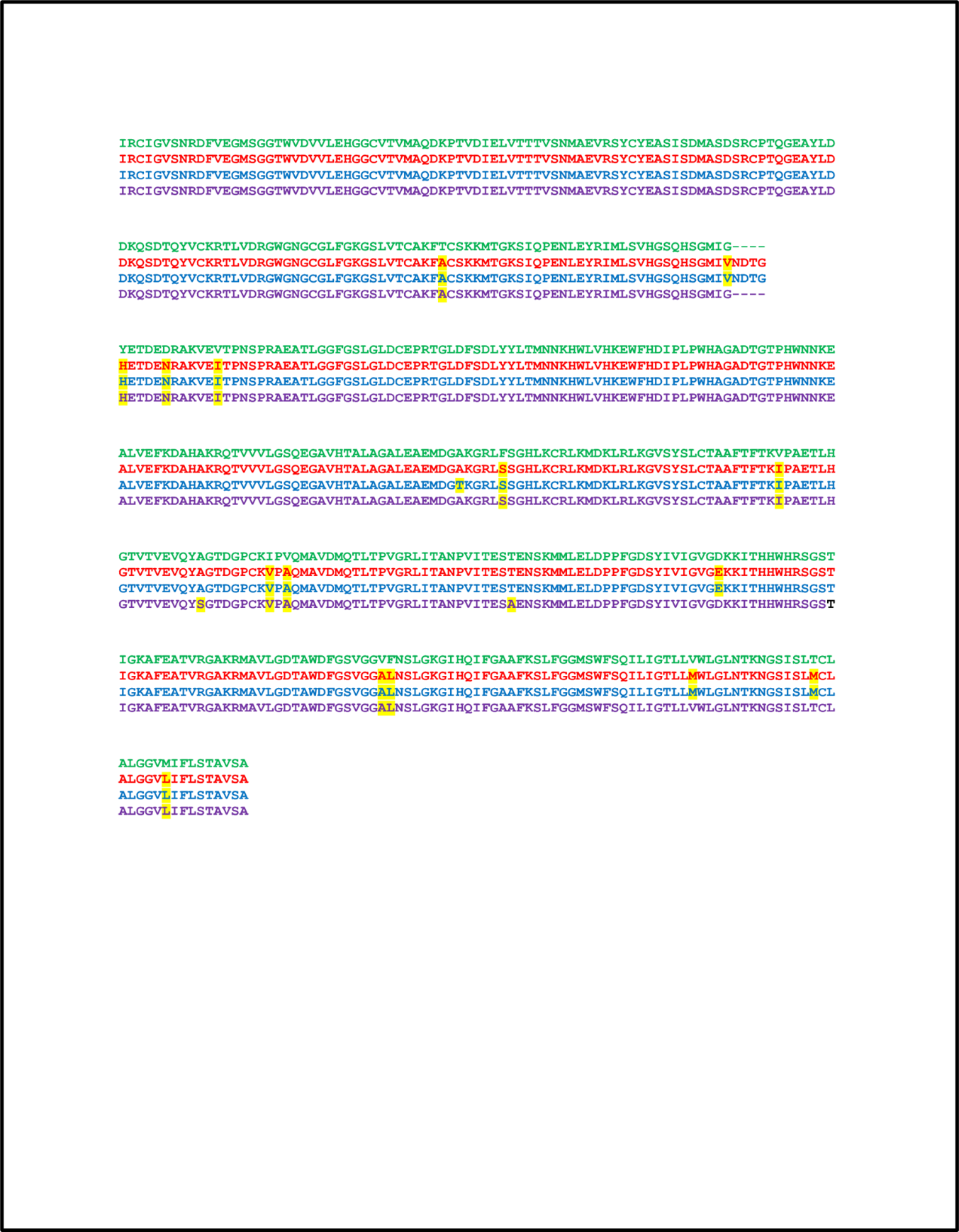

Supplement: Supplementary Figure 1 — Multiple sequence alignment of E protein in ZIKV strains: ZIKV MR766 (coloured in green), ZIKV NATAL RGN (coloured in red), ZIKV_RAJ (coloured in blue), and ZIKV_MAH (coloured in purple). All the mutations are highlighted in yellow with respect to ZIKV MR766. [file Image1.tif]

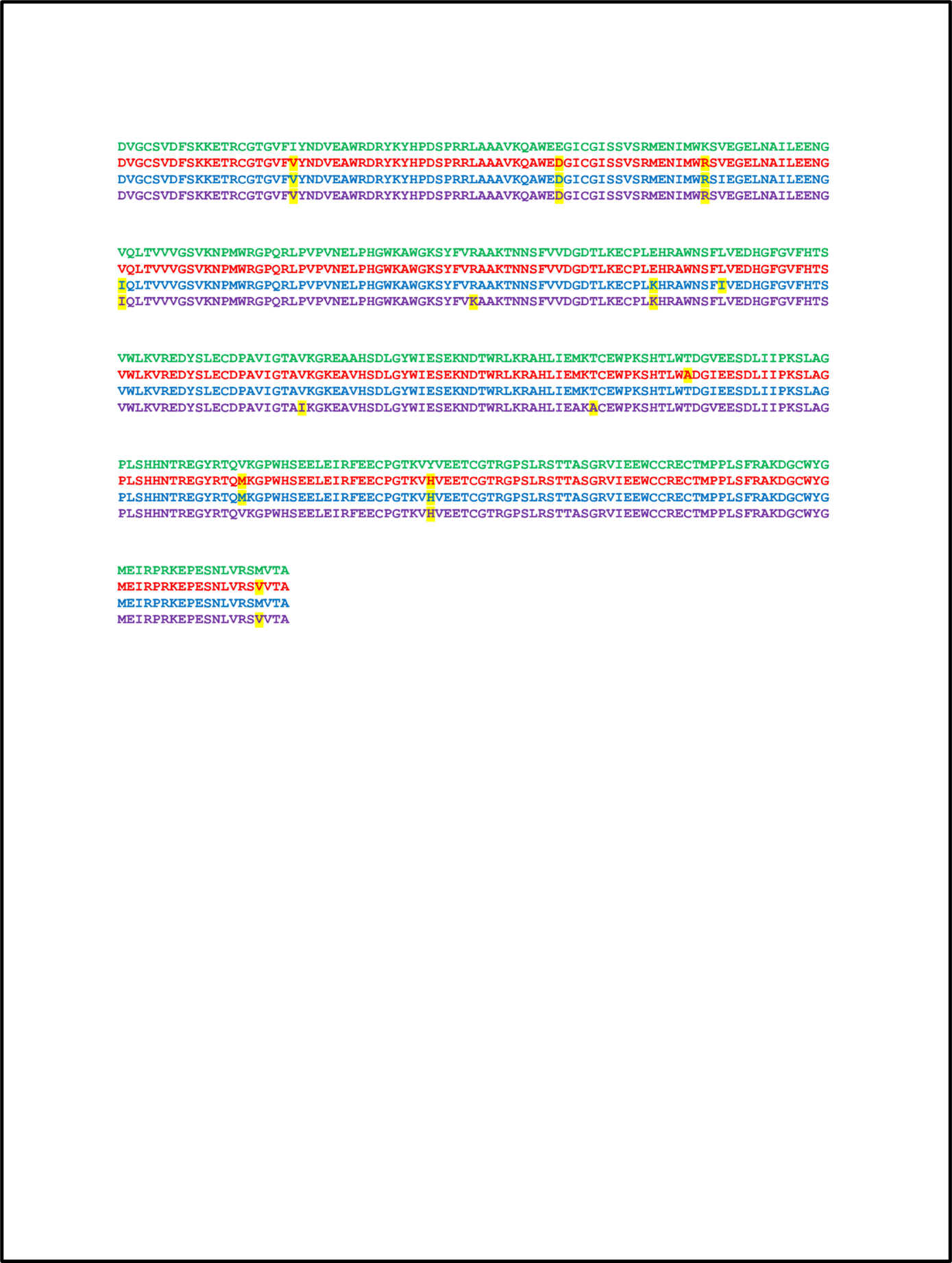

Supplement: Supplementary Figure 2 — Multiple sequence alignment of NS1 protein in ZIKV strains; ZIKV MR766 (coloured in green), ZIKV NATAL RGN (coloured in red), ZIKV_RAJ (coloured in blue), and ZIKV_MAH (coloured in purple). All the mutations are highlighted in yellow with respect to ZIKV MR766. [file Image2.tif]

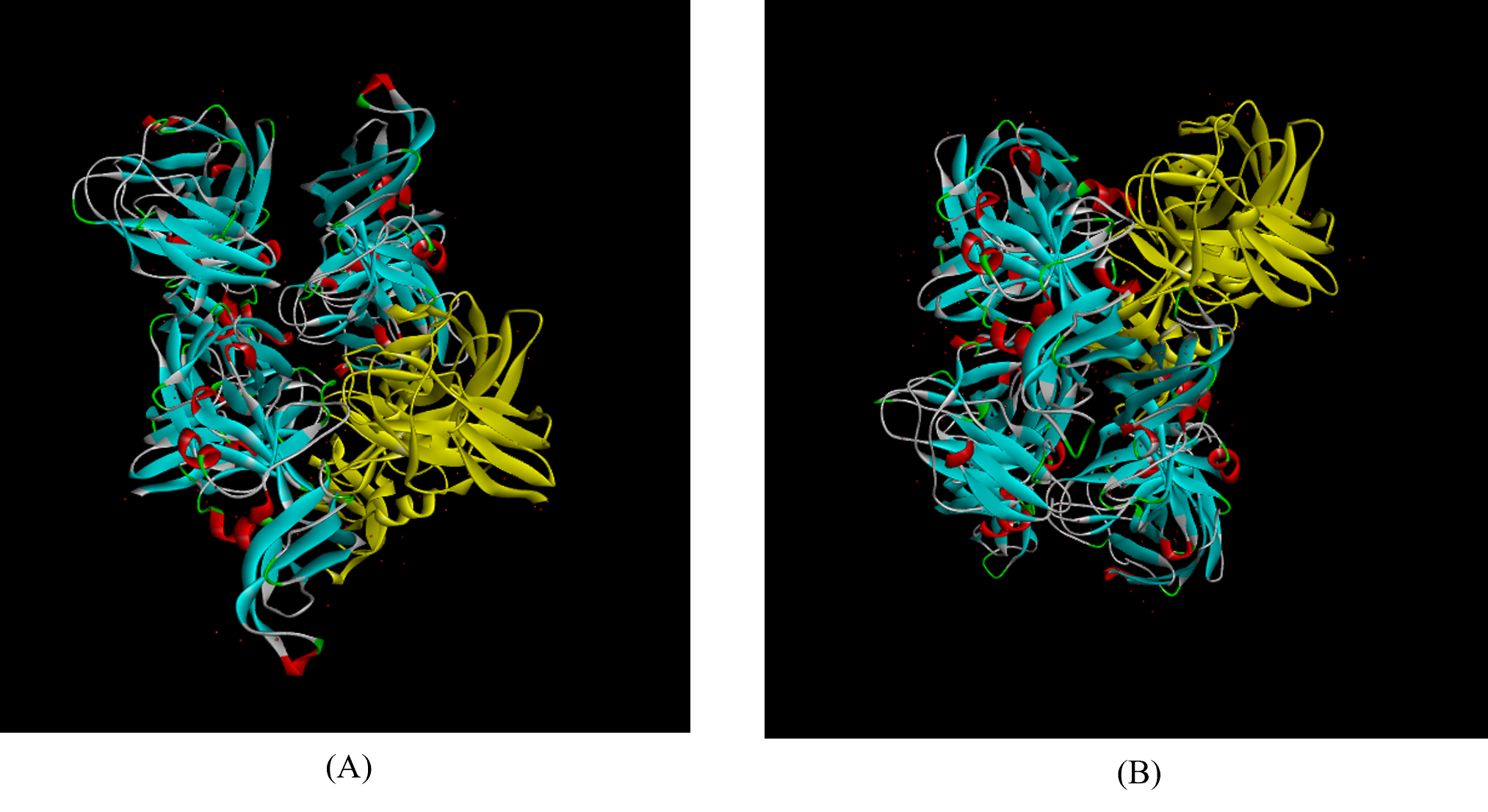

Supplement: Supplementary Figure 3 — 3-D Templates for Indian ZIKV E; (A) 7YW8 (ZIKV_RAJ), (B) 7YW7 (ZIKV_MAH) where Chain A is highlighted in yellow. [file Image3.tif]

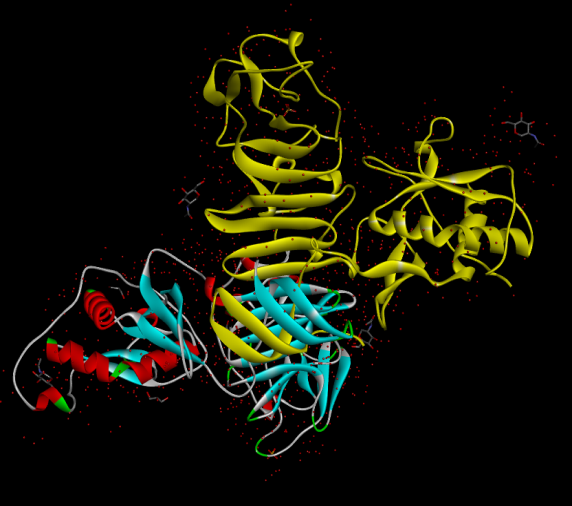

Supplement: Supplementary Figure 4 — 3-D Template for Indian ZIKV NS1: 5K6K (ZIKV_RAJ and ZIKV_MAH) where Chain A is highlighted in yellow. [file Image4.tif]

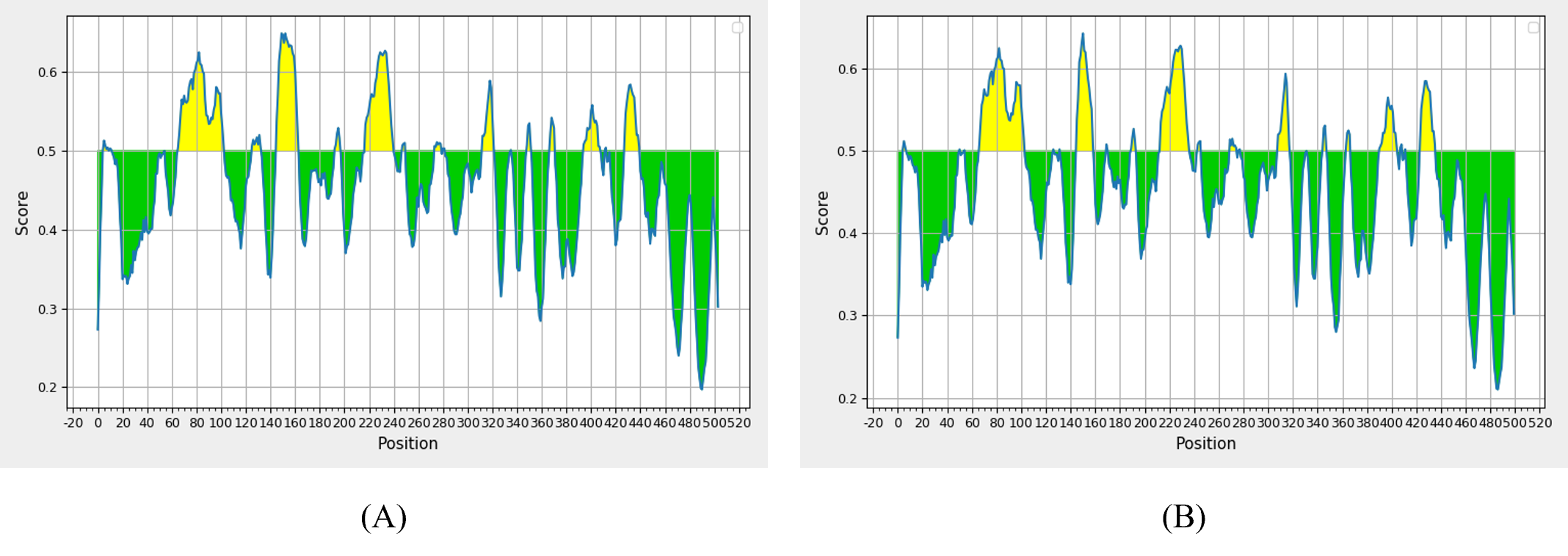

Supplement: Supplementary Figure 5 — Graphical representation of linear B-cell epitopes by BepiPred 2.0 method (yellow peaks = predicted epitopes, and green inverted peaks = non-epitopes) for both Indian ZIKV E (Threshold=0.5). (A) ZIKV_RAJ and (B) ZIKV_MAH. [file Image5.tif]

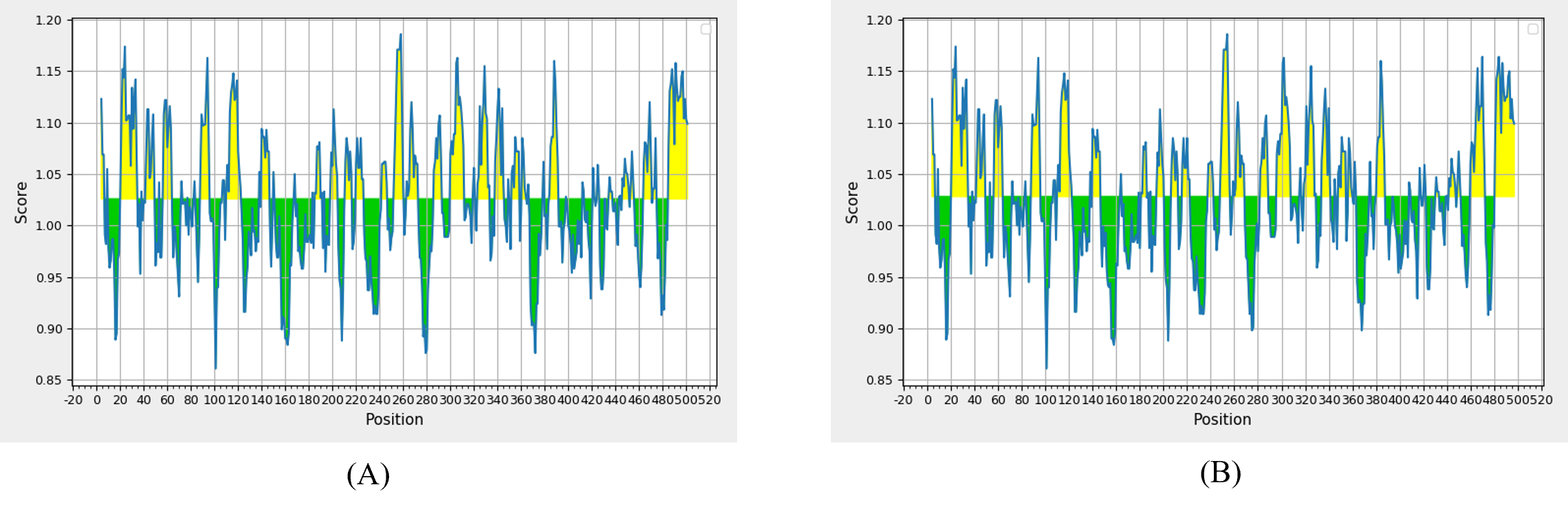

Supplement: Supplementary Figure 6 — Graphical representation of linear B-cell epitopes by Kolaskar and Tongaonkar method (yellow peaks = predicted epitopes, and green inverted peaks = non-epitopes) for both Indian ZIKV E. (A) ZIKV_RAJ (Threshold=1.026) and (B) ZIKV_MAH (Threshold=1.028). [file Image6.tif]

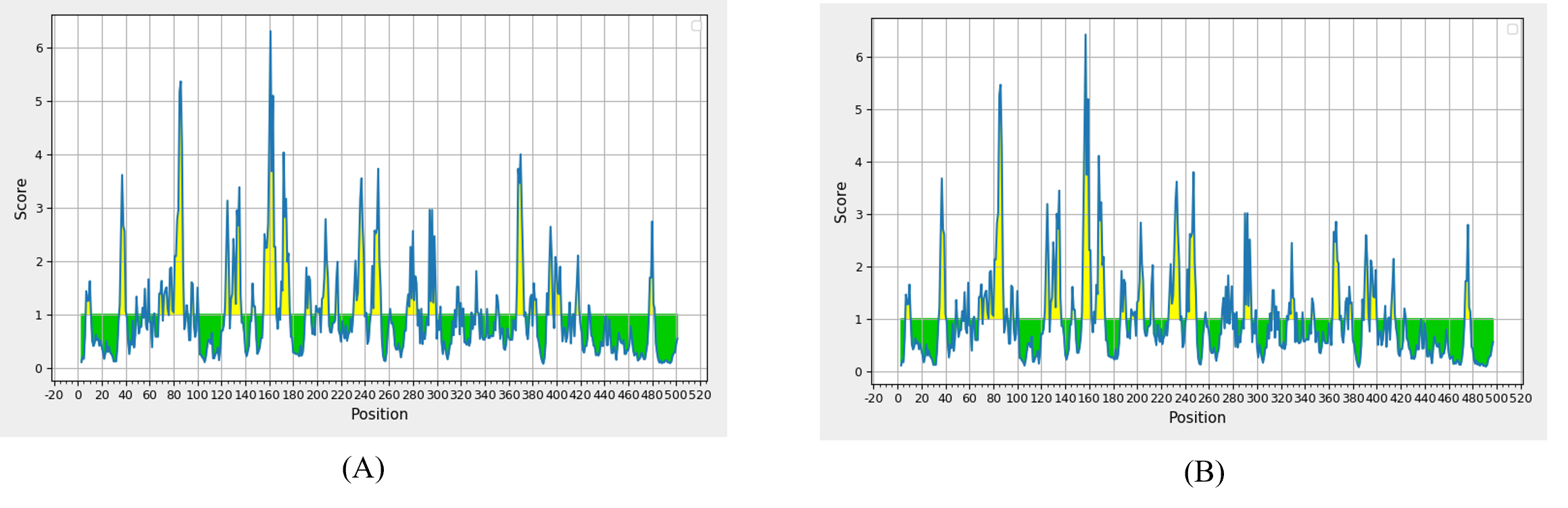

Supplement: Supplementary Figure 7 — Graphical representation of linear B-cell epitopes by Emini surface accessibility method (yellow peaks = predicted epitopes, and green inverted peaks = non-epitopes) for both Indian ZIKV E (Threshold=1.00). (A) ZIKV_RAJ and (B) ZIKV_MAH. [file Image7.tif]

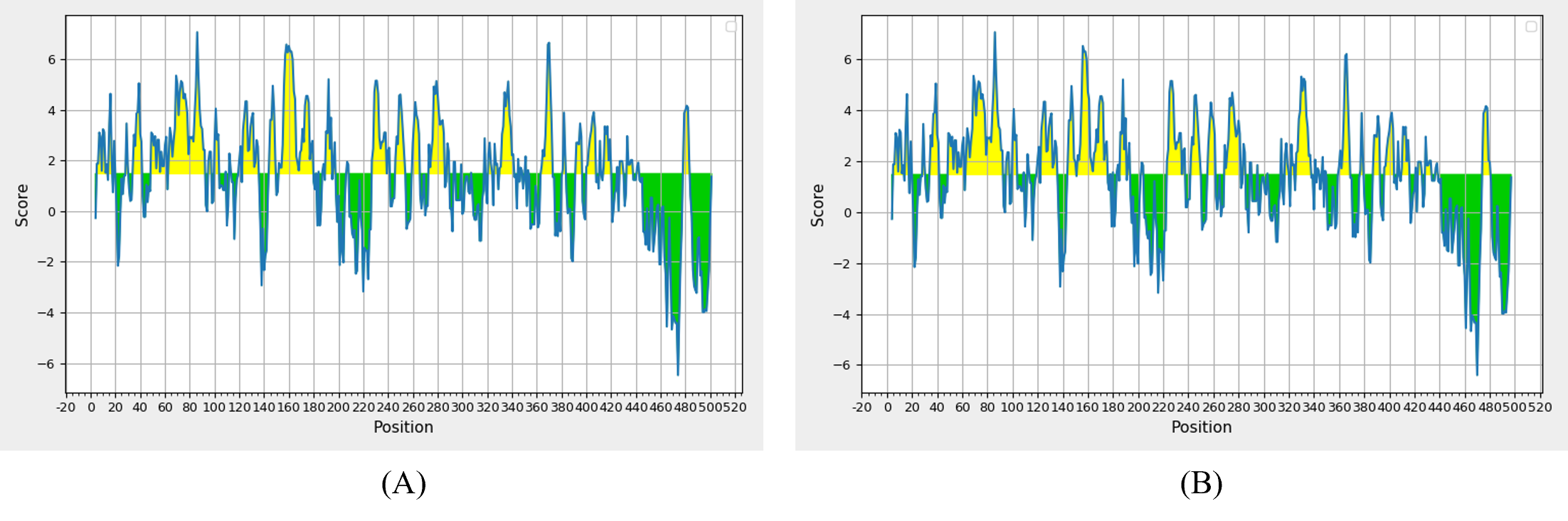

Supplement: Supplementary Figure 8 — Graphical representation of linear B-cell epitopes by Parker hydrophilicity method (yellow peaks = predicted epitopes, and green inverted peaks = non-epitopes) for both Indian ZIKV E. (A) ZIKV_RAJ (Threshold=1.475) and (B) ZIKV_MAH (Threshold=1.470). [file Image8.tif]

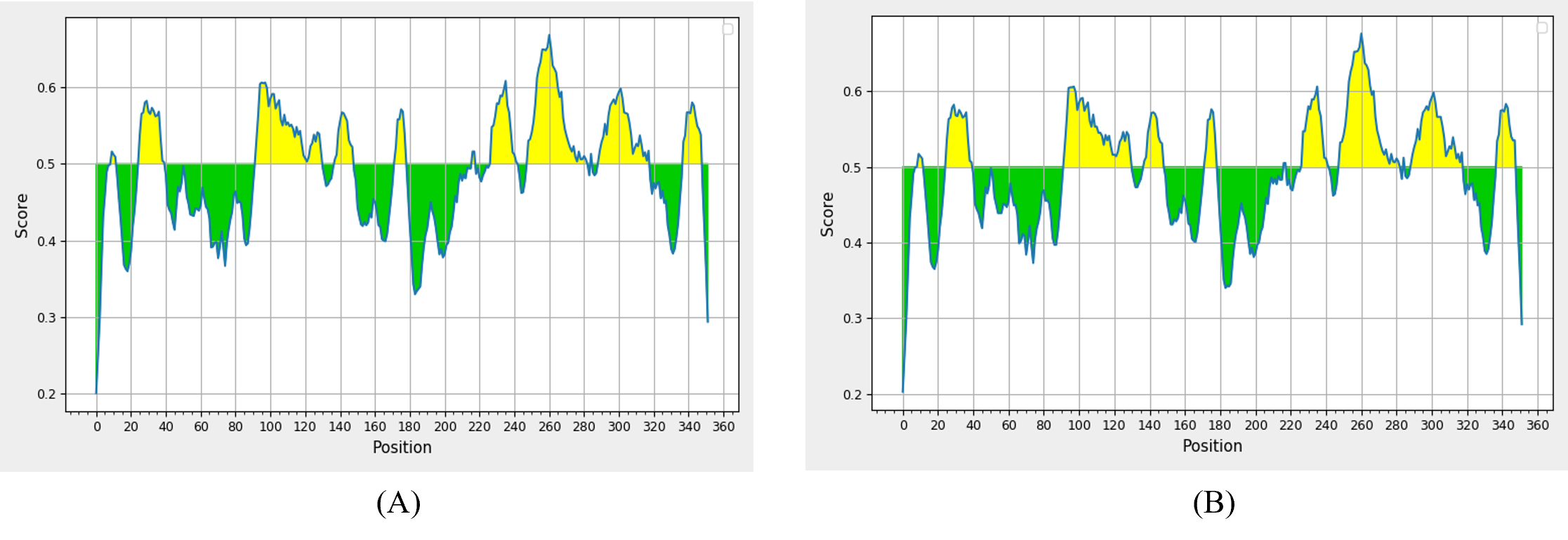

Supplement: Supplementary Figure 9 — Graphical representation of linear B-cell epitopes by BepiPred 2.0 method (yellow peaks = predicted epitopes, and green inverted peaks = non-epitopes) for both Indian ZIKV NS1 (Threshold=0.5). (A) ZIKV_RAJ and (B) ZIKV_MAH. [file Image9.tif]

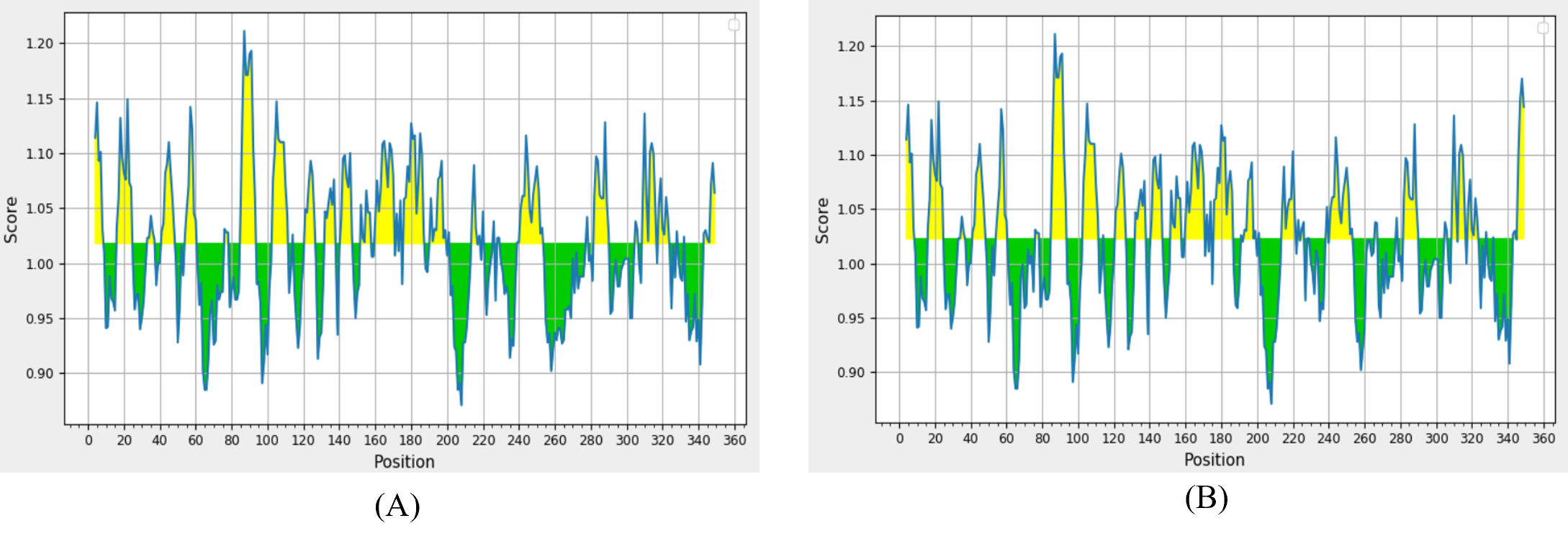

Supplement: Supplementary Figure 10 — Graphical representation of linear B-cell epitopes by Kolaskar and Tongaonkar method (yellow peaks = predicted epitopes, and green inverted peaks = non-epitopes) for both Indian ZIKV NS1. (A) ZIKV_RAJ (Threshold=1.018) and (B) ZIKV_MAH (Threshold=1.023). [file Image10.tif]

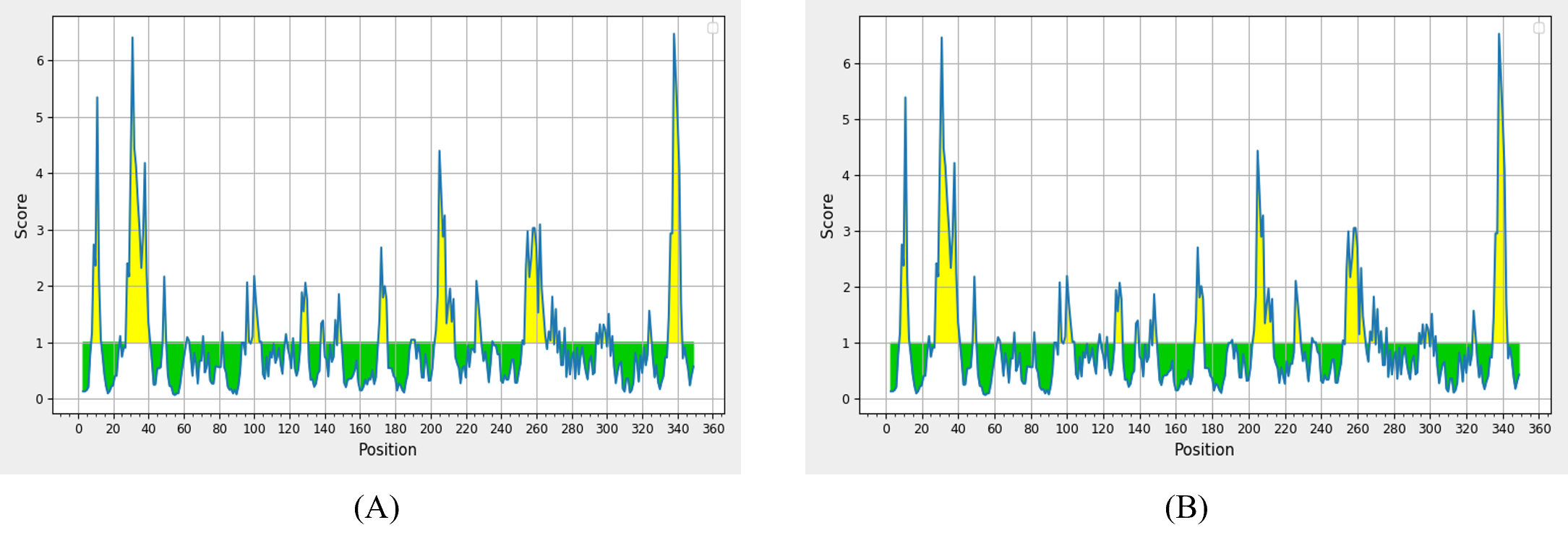

Supplement: Supplementary Figure 11 — Graphical representation of linear B-cell epitopes by Emini surface accessibility method (yellow peaks = predicted epitopes, and green inverted peaks = non-epitopes) for both Indian ZIKV NS1 (Threshold=1.00). (A) ZIKV_RAJ and (B) ZIKV_MAH. [file Image11.tif]

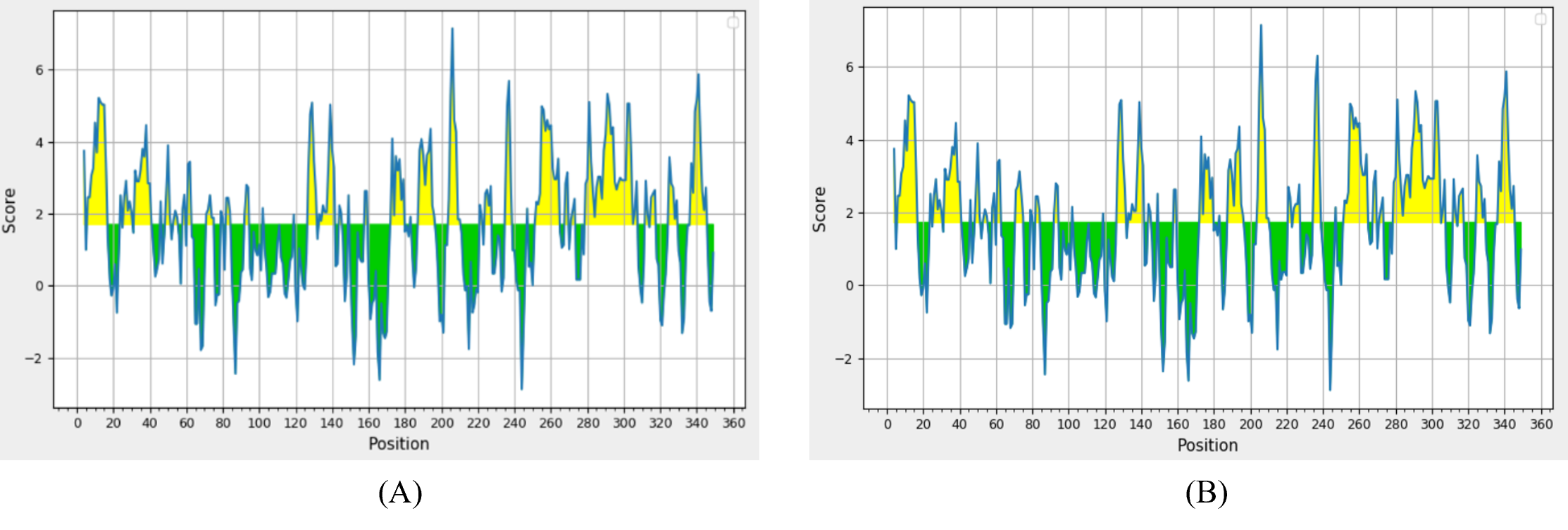

Supplement: Supplementary Figure 12 — Graphical representation of linear B-cell epitopes by Parker hydrophilicity method (yellow peaks = predicted epitopes, and green inverted peaks = non-epitopes) for both Indian ZIKV NS1. (A) ZIKV_RAJ (Threshold=1.701) and (B) ZIKV_MAH (Threshold=1.726). [file Image12.tif]
