# Supplementary material for: Identification of B-cell epitopes of Indian Zika virus strains using immunoinformatics
Source: Front Immunol. 2025 Feb 27;16:1534737. doi: 10.3389/fimmu.2025.1534737 (PMC11903408; doi:10.3389/fimmu.2025.1534737)
Supplement: Supplementary file 13 [file Table1.docx]

Table S1: Antigenicity scores of ZIKV E-specific domains

| ZIKV E-specific domains | Mapped Regions on E | Antigenicity scores (ZIKV MR766) | Antigenicity scores (ZIKV_RAJ) | Antigenicity scores (ZIKV_MAH) | Antigenicity scores (ZIKV NATAL RGN) |
| --- | --- | --- | --- | --- | --- |
| EDI (E domain I) | 1-51 | 0.7414 | 0.7414 | 0.7414 | 0.7414 |
|  | 132-192 | 0.8065 | 0.7269 | 0.8373 | 0.7269 |
|  | 280-295 | 0.5630 | 1.1801 | 1.0892 | 1.0892 |
| EDII (E domain II) | 52-132 | 0.4075 | 0.3934* | 0.3934* | 0.3934* |
|  | 193-279 | 0.7196 | 0.7196 | 0.7196 | 0.7196 |
| EDIII (E domain III) | 296-403 | 0.5302 | 0.5162 | 0.5195 | 0.5162 |

Threshold: 0.4; *Indicates antigenicity values below 0.4
