# Supplementary material for: Identification of B-cell epitopes of Indian Zika virus strains using immunoinformatics
Source: Front Immunol. 2025 Feb 27;16:1534737. doi: 10.3389/fimmu.2025.1534737 (PMC11903408; doi:10.3389/fimmu.2025.1534737)
Supplement: Supplementary file 14 [file Table2.docx]

Table S2: Antigenicity scores of ZIKV NS1-specific domains

| ZIKV NS1-specific domains | Mapped Regions  on NS1 | Antigenicity scores (ZIKV MR766) | Antigenicity scores (ZIKV_RAJ) | Antigenicity scores (ZIKV_MAH) | Antigenicity scores (ZIKV NATAL RGN) |
| --- | --- | --- | --- | --- | --- |
| Beta Roll | 1-29 | 0.6824 | 0.7338 | 0.7338 | 0.7338 |
| Wing Domain (Wing Flexible loop: 108-129 and Greasy Finger: 159-163) | 30-180 | 0.2332* | 0.2249* | 0.2398* | 0.2382* |
| Beta Ladder  (Spaghetti Loop: 219-272) | 181-352 | 0.5708 | 0.5768 | 0.5932 | 0.5898 |

Threshold: 0.4; *Indicates antigenicity values below 0.4
