# Supplementary material for: Identification of B-cell epitopes of Indian Zika virus strains using immunoinformatics
Source: Front Immunol. 2025 Feb 27;16:1534737. doi: 10.3389/fimmu.2025.1534737 (PMC11903408; doi:10.3389/fimmu.2025.1534737)
Supplement: Supplementary file 15 [file Table3.docx]

Table S3: ABCpred linear B-cell epitope predictions for Indian ZIKV E

| **ZIKV_RAJ-Specific Epitopes** | **Score** | **Rank** | **ZIKV_MAH-Specific epitopes** | **Score** | **Rank** |
| --- | --- | --- | --- | --- | --- |
| 327-TVEVQYAGTDGPCKVP-342  165-AKVEITPNSPRAEATL-180  183-FGSLGLDCEPRTGLDF-198  156-TGHETDENRAKVEITP-171  149-SGMIVNDTGHETDENR-164  32-TVMAQDKPTVDIELVT-47  216-EWFHDIPLPWHAGADT-231  61-YEASISDMASDSRCPT-76  305-YSLCTAAFTFTKIPAE-320  118-KFACSKKMTGKSIQPE-133  282-GRLSSGHLKCRLKMDK-297  224-PWHAGADTGTPHWNNK-239  17-GGTWVDVVLEHGGCVT-32  99-RGWGNGCGLFGKGSLV-114  422-AVLGDTAWDFGSVGGA-437  392-GEKKITHHWHRSGSTI-407  356-GRLITANPVITESTEN-371  464-SQILIGTLLMWLGLNT-479  43-IELVTTTVSNMAEVRS-58  362-NPVITESTENSKMMLE-377  197-DFSDLYYLTMNNKHWL-212  416-RGAKRMAVLGDTAWDF-431  5-GVSNRDFVEGMSGGTW-20  128-KSIQPENLEYRIMLSV-143  78-GEAYLDKQSDTQYVCK-93  69-ASDSRCPTQGEAYLDK-84  374-MMLELDPPFGDSYIVI-389  333-AGTDGPCKVPAQMAVD-348 | 0.94  0.94  0.91  0.90  0.89  0.88  0.88  0.87  0.87  0.87  0.86  0.86  0.86  0.85  0.85  0.84  0.84  0.83  0.83  0.83  0.83  0.82  0.81  0.81  0.80  0.80  0.80  0.80 | 1  1  2  3  4  5  5  6  6  6  7  7  7  8  8  9  10  11  11  11  11  12  13  13  14  14  14  14 | 161-AKVEITPNSPRAEATL-176  323-TVEVQYSGTDGPCKVP-338  179- FGSLGLDCEPRTGLDF-194  32- TVMAQDKPTVDIELVT-47  212- EWFHDIPLPWHAGADT-227  61- YEASISDMASDSRCPT-76  301- YSLCTAAFTFTKIPAE-316  148- HSGMIGHETDENRAKV-163  118- KFACSKKMTGKSIQPE-133  388- GDKKITHHWHRSGSTI-403  278- GRLSSGHLKCRLKMDK-293  220- PWHAGADTGTPHWNNK-235  17-GGTWVDVVLEHGGCVT-32  99- RGWGNGCGLFGKGSLV-114  418- AVLGDTAWDFGSVGGA-433  352-GRLITANPVITESAEN-367  43- IELVTTTVSNMAEVRS-58  193-DFSDLYYLTMNNKHWL-208  412- RGAKRMAVLGDTAWDF-427  5-GVSNRDFVEGMSGGTW-20  455-GMSWFSQILIGTLLVW-470  128-KSIQPENLEYRIMLSV-143  78-GEAYLDKQSDTQYVCK-93  69- ASDSRCPTQGEAYLDK-85  370-MMLELDPPFGDSYIVI-386 | 0.94  0.92  0.91  0.88  0.88  0.87  0.87  0.87  0.87  0.86  0.86  0.86  0.86  0.85  0.85  0.85  0.83  0.83  0.82  0.81  0.81  0.81  0.80  0.80  0.80 | 1  2  3  4  4  5  5  5  5  6  6  6  6  7  7  7  8  8  9  10  10  10  11  11  11 |

ZIKV_RAJ: Left and ZIKV_MAH: Right
