# Supplementary material for: Identification of B-cell epitopes of Indian Zika virus strains using immunoinformatics
Source: Front Immunol. 2025 Feb 27;16:1534737. doi: 10.3389/fimmu.2025.1534737 (PMC11903408; doi:10.3389/fimmu.2025.1534737)
Supplement: Supplementary file 16 [file Table4.docx]

Table S4: BepiPred 2.0 linear B-cell epitope predictions for Indian ZIKV E

| **ZIKV_RAJ-Specific Epitopes** | **ZIKV_MAH-Specific Epitopes** |
| --- | --- |
| 5-GVSNR-9  11-FV-12  55-E-55  66-SDMASDSRCPTQGEAYLDKQSDTQYVCKRTLVDRGWN-103  126-TGKSIQPE-133  146-SQHSGMIVNDTGHETDEN-163  193-RTGLD-197  218-FHDIPLPWHAGADTGTPHWNNKE-240  248-AHA-250  274-EAEMDG-279  281-KG-282  313-TFTKIPAETL-322  336-D-336  349-MQTL-352  368-STEN-371  395-KITHHWHRSGSTIG-408  413-A-413  428-AWDFGSVGGALNS-440 | 5-GVS-7  51-S-51  55-E-55  67-DMASDSRCPTQGEAYLDKQSDTQYVCKRTLVDRGWGN-103  127-G-127  132-P-132  146-SQHSGMIGHETDEN-159  170-P-170  190-TGLD-193  214-FHDIPLPWHAGADTGTPHWNNKEA-237  244-AHA-246  270-EAEMDG-275  277-KG-278  309-TFTKIPAETL-318  322-D-322  345-MQTL-348  364-SAEN-367  391-KITHHWHRSGSTIGK-405  408-EA-409  412-R-412  424-AWDFGSVGGALNS-436 |

ZIKV_RAJ: Left and ZIKV_MAH: Right.
