# Supplementary material for: Identification of B-cell epitopes of Indian Zika virus strains using immunoinformatics
Source: Front Immunol. 2025 Feb 27;16:1534737. doi: 10.3389/fimmu.2025.1534737 (PMC11903408; doi:10.3389/fimmu.2025.1534737)
Supplement: Supplementary file 17 [file Table5.docx]

Table S5: Kolaskar and Tongaonkar linear B-cell epitope predictions for Indian ZIKV E

| **ZIKV_RAJ-specific epitopes** | **ZIKV_MAH-specific epitopes** |
| --- | --- |
| 20-WVDVVLEHGGCVTVM-34  42-DIELVTTT-49  56-VRSYCYEA-63  88-TQYVCKRT-95  110-KGSLVTCAKFACS-122  140-MLSVHGS-146  185-SLGLDCE-191  198-FSDLYYL-204  211-WLVHKE-216  220-DIPLPWH-226  252-RQTVVVLGS-260  263-GAVHTALAGA-272  286-SGHLKCR-292  300-LKGVSYSLCTAA-311  323-HGTVTVEVQYA-333  338-PCKVPAQM-345  352-LTPVGRL-358  384-DSYIVIGV-391  446-HQIFGA-451  460-SQILIGT-470 | 21-VDVVLEHGGCVTVM-34  42-DIELVTTT-49  57-RSYCYEA-63  88-TQYVCKRT-95  110-KGSLVTCAKFACS-122  140-MLSVHGS-146  194-FSDLYYL-200  207-WLVHKE-212  216-DIPLPWH-222  248-RQTVVVLGS-256  259-GAVHTALAGA-268  282-SGHLKCR-288  296-LKGVSYSLCTAA-307  319-HGTVTVEVQYS-329  334-PCKVPAQM-341  348-LTPVGRL-354  380-DSYIVIGV-387  442-HQIFGA-447  460-SQILIGTLLVWLG-472 |

ZIKV_RAJ: Left and ZIKV_MAH: Right
