# Supplementary material for: Identification of B-cell epitopes of Indian Zika virus strains using immunoinformatics
Source: Front Immunol. 2025 Feb 27;16:1534737. doi: 10.3389/fimmu.2025.1534737 (PMC11903408; doi:10.3389/fimmu.2025.1534737)
Supplement: Supplementary file 18 [file Table6.docx]

Table S6: ABCpred linear B-cell epitope predictions for Indian ZIKV NS1

| **ZIKV_RAJ-Specific Epitopes** | Score | Rank | **ZIKV_MAH-Specific Epitopes** | Score | Rank |
| --- | --- | --- | --- | --- | --- |
| 314-RECTMPPLSFRAKDGC-329  260-YRTQMKGPWHSEELEI-275  81-ENGIQLTVVVGSVKNP-96  248-AGPLSHHNTREGYRTQ-263  11-KETRCGTGVFVYNDVE-26  187-AVKGKEAVHSDLGYWI-202  229-HTLWTDGIEESDLIIP-244  90-VGSVKNPMWRGPQRLP-105  22-YNDVEAWRDRYKYHPD-37  193-AVHSDLGYWIESEKND-208  147-HRAWNSFIVEDHGFGV-162  331-YGMEIRPRKEPESNLV-346  305-GRVIEEWCCRECTMPP-320  298-LRSTTASGRVIEEWCC-314  202-IESEKNDTWRLKRAHL-217  181-PAVIGTAVKGKEAVHS-196  138-DTLKECPLKHRAWNSF-153  117-AWGKSYFVRAAKTNNS-132  30-DRYKYHPDSPRRLAAA-45  285-VHVEETCGTRGPSLRS-300  54-ICGISSVSRMENIMWR-69  47-KQAWEDGICGISSVSR-62  170-KVREDYSLECDPAVIG-185  215-AHLIEMKTCEWPKSHT-230 | 0.94  0.92  0.91  0.91  0.91  0.89  0.88  0.87  0.87  0.87  0.86  0.85  0.85  0.84  0.84  0.84  0.84  0.84  0.83  0.83  0.82  0.82  0.82  0.81 | 1  2  3  3  3  4  5  6  6  6  7  8  8  9  9  9  9  9  10  10  11  11  11  12 | 314-RECTMPPLSFRAKDGC-329  81-ENGIQLTVVVGSVKNP-96  248-AGPLSHHNTREGYRTQ-263  11-KETRCGTGVFVYNDVE-26  260-YRTQVKGPWHSEELEI-275  229-HTLWTDGVEESDLIIP-244  147-HRAWNSFLVEDHGFGV-162  90-VGSVKNPMWRGPQRLP-105  22-YNDVEAWRDRYKYHPD-37  193-AVHSDLGYWIESEKND-208  187-AIKGKEAVHSDLGYWI-202  331-YGMEIRPRKEPESNLV-346  305-GRVIEEWCCRECTMPP-320  121-SYFVKAAKTNNSFVVD-136  114-GWKAWGKSYFVKAAKT-129  298-LRSTTASGRVIEEWCC-313  202-IESEKNDTWRLKRAHL-217  138-DTLKECPLKHRAWNSF-153  30-DRYKYHPDSPRRLAAA-45  285-VHVEETCGTRGPSLRS-300  181-PAVIGTAIKGKEAVHS-196  54-ICGISSVSRMENIMWR-69  47-KQAWEDGICGISSVSR-62  170-KVREDYSLECDPAVIG-185  235-GVEESDLIIPKSLAGP-250  215-AHLIEAKACEWPKSHT-230 | 0.94  0.91  0.91  0.91  0.90  0.89  0.89  0.87  0.87  0.87  0.87  0.85  0.85  0.85  0.85  0.84  0.84  0.84  0.83  0.83  0.83  0.82  0.82  0.82  0.80  0.80 | 1  2  2  2  3  4  4  5  5  5  5  6  6  6  6  7  7  7  8  8  8  9  9  9  10  10 |

ZIKV_RAJ: Left and ZIKV_MAH: Right
