# Supplementary material for: Identification of B-cell epitopes of Indian Zika virus strains using immunoinformatics
Source: Front Immunol. 2025 Feb 27;16:1534737. doi: 10.3389/fimmu.2025.1534737 (PMC11903408; doi:10.3389/fimmu.2025.1534737)
Supplement: Supplementary file 19 [file Table7.docx]

Table S7: BepiPred 2.0 linear B-cell epitope predictions for Indian ZIKV NS1

| **ZIKV_RAJ-Specific Epitopes** | **ZIKV_MAH-Specific Epitopes** |
| --- | --- |
| 10-KKE-12  26-EAWRDRYKYHPDSPR-40  93-VKNPMWRGPQRLPVPVNELPHGWKAWGKSYFVRAAKTN-130  138-DTLKECPLKHR-148  172-REDYSLE-178  217-LI-218  227-KSHTLWTDGIEESDLI-242  249-GPLSHHNTREGYRTQMKGPWHSEELEIRFEECPG-282  285-V  289-ETCGTRGPSLRSTTASGRVIEEWCCRECTM-318  338-RKEPESNLVRS-348 | 9-SKKE-12  26-EAWRDRYKYHPDSPR-40  93-VKNPMWRGPQRLPVPVNELPHGWKAWGKSYFVKAAKTN-130  138-DTLKECPLKHR-148  172-REDYSLEC-179  217-LI-218  227-KSHTLWTDGVEESDLI-242  249-GPLSHHNTREGYRTQVKGPWHSEELEIRFEECPG-282  285-V  289-ETCGTRGPSLRSTTASGRVIEEWCCRECTM-318  338-RKEPESNLVRS-348 |

ZIKV_RAJ: Left and ZIKV_MAH: Right
