# Supplementary material for: Identification of B-cell epitopes of Indian Zika virus strains using immunoinformatics
Source: Front Immunol. 2025 Feb 27;16:1534737. doi: 10.3389/fimmu.2025.1534737 (PMC11903408; doi:10.3389/fimmu.2025.1534737)
Supplement: Supplementary file 20 [file Table8.docx]

Table S8: Kolaskar and Tongaonkar linear B-cell epitope predictions for Indian ZIKV NS1

| **ZIKV_RAJ-Specific Epitopes** | **ZIKV_MAH-Specific Epitopes** |
| --- | --- |
| 16-GTGVFVYND-24  41-RLAAAVKQ-48  54-ICGISSV-60  85-QLTVVVGSV-93  103-RLPVPVNEL-111  121-SYFVRA-126  132-SFVVDG-137  141-KECPLKH-147  152-SFIVED-157  160-FGVFHTSVWLK-170  176-SLECDPAVIGTA-187  191-KEAVHSDLG-199  239-SDLIIPKSLAGPLSH-253  282-GTKVHVEET-290  309-EEWCCREC-316  318-MPPLSF-323 | 16-GTGVFVYND-24  41-RLAAAVKQ-48  54-ICGISSV-60  85-QLTVVVGSV-93  103-RLPVPVNEL-111  121-SYFVRA-126  132-SFVVDG-137  141-KECPLKH-147  152-SFIVED-157  160-FGVFHTSVWLK-170  176-SLECDPAVIGT-186  193-AVHSDLG-199  214-RAHLIEAK-221  241-LIIPKSLAGPLSH-253  282-GTKVHVEET-290  318-MPPLSF-323 |

ZIKV_RAJ: Left and ZIKV_MAH: Right
