# Supplementary material for: Identification of B-cell epitopes of Indian Zika virus strains using immunoinformatics
Source: Front Immunol. 2025 Feb 27;16:1534737. doi: 10.3389/fimmu.2025.1534737 (PMC11903408; doi:10.3389/fimmu.2025.1534737)
Supplement: Supplementary file 21 [file Table9.docx]

Table S9: List of predicted ZIKV E-specific conformational B-cell epitopes

| **Conformational B-cell Epitopes** | **ZIKV Strains** | **E Domain** |
| --- | --- | --- |
| **G383**, **H401**, D384, **S403**  W101 | ZIKV_RAJ  ZIKV_MAH | EDIII  EDII |

Novel epitopes are highlighted in Bold
