# Supplementary material for: Identification of B-cell epitopes of Indian Zika virus strains using immunoinformatics
Source: Front Immunol. 2025 Feb 27;16:1534737. doi: 10.3389/fimmu.2025.1534737 (PMC11903408; doi:10.3389/fimmu.2025.1534737)
Supplement: Supplementary file 22 [file Table10.docx]

Table S10: List of predicted ZIKV NS1-specific conformational B-cell epitopes

| **Conformational B-cell Epitopes** | **ZIKV Strains** | **NS1 Domain** |
| --- | --- | --- |
| **F8, S9, K10, K11**  **K116, A117, W118, G119, K120**  A303, S304, G305, **P341, S343**  **F8, S9, K10, K11**  A303**, S343** | ZIKV_RAJ  ZIKV_MAH | β-Roll  Wing Domain  β-Ladder  β-Roll  β-Ladder |

Novel epitopes are Highlighted in bold.
